# Construction and Optimization of the *de novo* Biosynthesis Pathway of Mogrol in *Saccharomyces Cerevisiae*

**DOI:** 10.3389/fbioe.2022.919526

**Published:** 2022-05-27

**Authors:** Siyu Wang, Xianhao Xu, Xueqin Lv, Yanfeng Liu, Jianghua Li, Guocheng Du, Long Liu

**Affiliations:** ^1^ Key Laboratory of Carbohydrate Chemistry and Biotechnology, Ministry of Education, Jiangnan University, Wuxi, China; ^2^ Science Center for Future Foods, Ministry of Education, Jiangnan University, Wuxi, China

**Keywords:** *Siraitia grosvenorii*, Mogrol, *S. cerevisiae*, CRISPRi, Metabolic Engineering

## Abstract

Mogrol plays important roles in antihyperglycemic and antilipidemic through activating the AMP-activated protein kinase pathway. Although the synthesis pathway of mogrol in *Siraitia grosvenorii* has been clarified, few studies have focused on improving mogrol production. This study employed a modular engineerin g strategy to improve mogrol production in a yeast chassis cell. First, a *de novo* synthesis pathway of mogrol in *Saccharomyces cerevisiae* was constructed*.* Then, the metabolic flux of each synthetic module in mogrol metabolism was systematically optimized, including the enhancement of the precursor supply, inhibition of the sterol synthesis pathway using the Clustered Regularly Interspaced Short Palindromic Repeats Interference system (CRISPRi), and optimization of the expression and reduction system of P450 enzymes. Finally, the mogrol titer was increased to 9.1 μg/L, which was 455-fold higher than that of the original strain. The yeast strains engineered in this work can serve as the basis for creating an alternative way for mogrol production in place of extraction from *S. grosvenorii*.

## Introduction

Mogrol, a cucurbitane-type tetracyclic triterpenoid compound, is the aglycone involved in mogroside synthesis. Mogrosides are digested into mogrol *in vivo* (Chiu et al., 2013; Wang et al., 2019). Therefore, mogrol may be the main functional unit of mogrosides to exert pharmacological effects (Liu et al., 2018). Many studies have shown that mogrol can effectively reduce blood glucose to contribute to antihyperglycemia and antilipidemia by activating AMP-activated protein kinase (Harada et al., 2016; Wang H. et al., 2020; Liang et al., 2021). Further studies have identified that it could attenuate pulmonary fibrosis, memory impairment, neuroinflammation, and apoptosis (Chen et al., 2018; Wang J. et al., 2020; Liu et al., 2021). Additionally, mogrol can also suppress leukemic cell growth by inhibiting the extracellular signal-regulated kinase 1/2 (ERK1/2) and signal transducer and activator of transcription three pathways (STAT3) implicated in leukemia carcinogenesis (Liu et al., 2015). Therefore, mogrol has a good application prospect in medicine.

Mogrosides and mogrol are extracted from *Siraitia grosvenorii* fruit, but the yield is low (0.55%–0.65%) (EFSA Panel on Food Additives and Flavourings et al., 2019). Besides, the tedious extraction process, slow growth rate, and growth process of *S. grosvenorii* are vulnerable to environmental perturbations, further limiting the large-scale production efficiency of mogrol. Therefore, it is crucial to develop another method for efficiently synthesizing mogrol. Recently, the introduction of heterologous natural product biosynthesis pathways into microbial cell factories to achieve the high-efficiency synthesis of natural products is attracting the attention of researchers. The advantages of microbial synthesis of natural products include rapid growth rate, simple cell genetic manipulation, and product extraction process (Scalcinati et al., 2012; Kotopka et al., 2018; Li et al., 2018). Many natural products have been efficiently synthesized in microbial cell factories, such as protopanaxadiol (Zhao et al., 2016; Zhao et al., 2019), β-carotene (Araya et al., 2012; Bu et al., 2020), taxol (Engels et al., 2008; Ajikumar et al., 2010), and cannabinoids (Luo et al., 2019).

**FIGURE 1:**
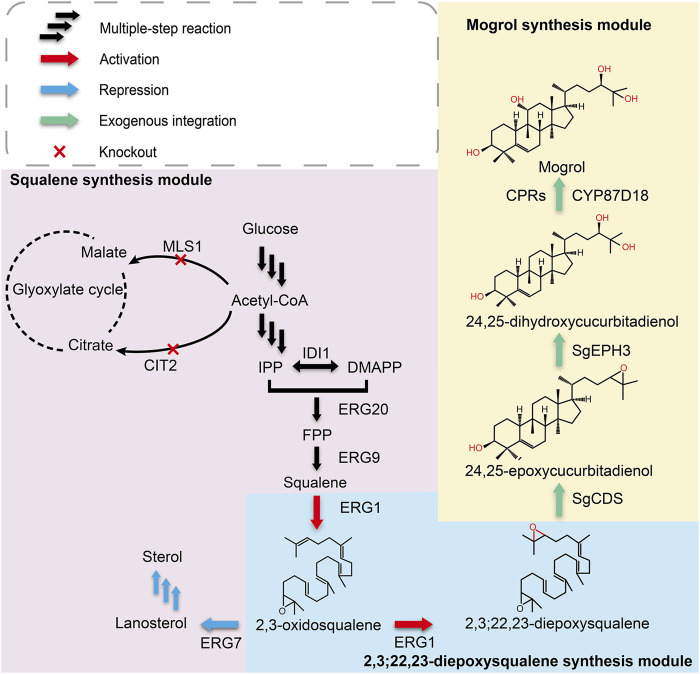
**Biosynthetic pathway for mogrol production in *S. cerevisiae.*** Single arrows represent one-step conversions, whereas triple arrows represent multiple steps. Overexpressed steps are shown as red arrows, suppressed steps are shown as blue arrows, and exogenous integrated steps are shown as green arrows. Abbreviations: IPP, isopentenyl pyrophosphate; DMAPP, dimethylallyl pyrophosphate; FPP, farnesyl; IDI1, isopentenyl diphosphate isomerase; ERG20, farnesyl pyrophosphate synthetase; ERG9, squalene synthase; ERG1, squalene epoxidase; ERG7, lanosterol synthase; MLS1, malate synthase; CIT2, citrate synthase; SgCDS, cucurbitadienol synthase; SgEPH3, epoxide hydrolase; CYP87D18, cytochrome P450 enzyme.

The biosynthesis pathway of mogrol has been extensively studied in recent years. Many studies have presumed that cucurbitadienol may be a precursor for mogrol synthesis. Therefore, a cucurbitadienol synthase gene (*SgCS*) from *S. grosvenorii* was heterologously expressed in *Saccharomyces cerevisiae*, and cucurbitadienol was detected in the fermentation broth, which realized the *de novo* biosynthesis of cucurbitadienol (Ta et al., 2012). However, the biosynthesis pathway from cucurbitadienol to mogrol has not been explored yet. Zhang et al. (2016) presumed that the enzyme CYP87D18 could catalyze the oxidation of cucurbitadienol at C-11 to generate 11-hydroxy cucurbitadienol, which could be a potential precursor to mogrol, but the following catalytic steps remained to be discovered. Therefore, researchers are committed to elucidating other pathways that could synthesize mogrol. Transcriptome and metabolomics are effective strategies to clarify the biosynthetic pathway of plant natural products (Tang et al., 2011; Dai et al., 2015). Itkin et al. (2016) screened a large number of candidate genes for mogrol biosynthesis according to the combination of genomic and transcriptomic profiling of *S. grosvenorii* and identified *SQE* (encoding squalene epoxidase), *CDS* (encoding cucurbitadienol synthase), *EPH* (encoding epoxide hydrolase), and *CYP* (encoding cytochrome P450-dependent monooxygenase) as the key genes for mogrol synthesis. Finally, Itkin et al. (2016) realized the *de novo* biosynthesis of mogrol in *S. cerevisiae* (Figure 1) and verified *in vitro* that UGT (glucosyltransferase) could catalyze mogrol to form mogrosides. However, the mogrol titer in their study was not quantified because it was still low.

In this study, *S. cerevisiae* was engineered as a chassis cell for mogrol biosynthesis. A modular engineering strategy was proposed to improve the synthesis efficiency of mogrol in chassis cells. A *de novo* biosynthesis pathway of mogrol was constructed in yeast by heterologously expressing *SgCDS*, *SgEPH3*, *CYP87D18*, and *AtCPR1* in strain Y4 (a high-yield squalene yeast constructed in our laboratory). *MLS1* (malate synthase; GenBank ID: 855606) and *CIT2* (citrate synthase; GenBank ID: 850361) were knocked out using the Clustered Regularly Interspaced Short Palindromic Repeats (CRISPRi) to enhance the precursor acetyl-CoA supply. The sterol synthesis pathway (essential for cell growth; Jordá and Puig, 2020) was repressed using CRISPRi to balance cell growth and production. The first step of the mogrol biosynthesis pathway was enhanced by overexpressing the *ERG1* gene, an important rate-limiting enzyme in the ergosterol synthesis pathway. Subsequently, cytochrome P450 reductases (CPRs) from various sources were codon-optimized and coexpressed with *CYP87D18* in the chassis cell to improve the catalysis efficiency of the last step of the mogrol biosynthesis pathway. Finally, by increasing the *CYP87D18* expression levels and paring with different CPRs, mogrol production reached 9.1 μg/L, which was 455-fold higher than that of the original strain. Overall, this study constructed a yeast platform strain for bioproduction of mogrol by systems metabolic engineering strategies.

## Materials and Methods

### Chemicals and Reagents

All chemicals were purchased from Sinopharm Chemical Reagent Co., Ltd. (Shanghai, China) unless otherwise specified. Mogrol standards were purchased from Sigma-Aldrich (St. Louis, MO, United States ). PrimeSTAR HS DNA polymerase used for target genes amplification was purchased from Takara (Japan). The GeneJET polymerase chain reaction (PCR) purification kit was purchased from Thermo Fisher Scientific (United States ), and SanPrep Spin Column & Collection Tube used for plasmid extraction was purchased from Sangon (Shanghai, China). The primers used in this study were synthesized by Genewiz (Suzhou, China). 5-Fluoroorotic acid (5-FOA), tyrosine, histidine, leucine, and uracil were purchased from Solarbio (Beijing, China).

### Strains and Medium

The strains and plasmids used in the study are listed in Table 1. *Escherichia coli* JM109 was used for constructing recombinant plasmids. *S. cerevisiae* Y4 was employed as the original strain, a high-yield strain of squalene constructed previously. All strains constructed in this study are listed in Table 1. *E. coli* was cultured in Luria–Bertani medium (10 g/L tryptone, 5 g/L yeast extract, and 10 g/L NaCl) containing 100 mg/L ampicillin at 37°C and 220 rpm, and yeast strains were cultured in YPD medium (10 g/L yeast extract, 20 g/L peptone, and 20 g/L glucose) at 30°C and 220 rpm. Yeast transformants were selected on synthetic dextrose (SD-Ura) agar plates containing 6.9 g/L yeast nitrogen base without amino acids (YNB medium), 20 g/L glucose, 0.1 g/L amino acids (l-leucine, l-tryptophan, and l-histidine), and 20 g/L agar. Dimethyl sulfoxide with 1 g/ml 5-FOA was used for the counterselection of recombinants containing the URA3 marker. G418 sulfate (200 mg/L; Sigma) was supplemented into the medium as required.

**TABLE 1:** Strains used in the study.

Strains	Characteristics	Resource
*E. coli* JM109	*recA*1, *endA*1, *thi, gyrA*96, *supE*44, *hsdR*17∆ (*lac*-*proAB*)/F’ [*traD*36, *proAB* ^+^, *lacІq*, *lacZ*∆ M15]	Lab stock
*S. cerevisiae* CEN.PK2-1C	*MATa ura3-52 trp1-289 leu2-3,112 his3D1 MAL2-8C SUC2*	Lab stock
Y4	CEN-PK2-1C derivate, Δ*ROX1*::*P*_*GPD*_-*tHMG1*-*TADH1*, inserting *P*_*TEF1*_-*IDI*-*T*_*CYC1*_-*P*_*GPD*_-*tHMG1*-*T*_*ADH1*_ and *P*_*TEF1*_-*ERG20*-*T*_*CYC1*_-*P*_*PGK1*_-*INO2*-*T*_*INO2*_ cassette	Lab stock
MOR001	Y4 derivate, inserting *T*_*ADH1*_-*SgCDS*-*PGAL1,10*-*SgEPH3*-*T*_*CYC1*_ and *T*_*TDH3*_-*CYP87D18*-*P*_*GAL1,10*_-*AtCPR1*-*T*_*CYC1*_ cassette	This work
MOR002	MOR001 derivate, Δ*CIT2* Δ*MLS1*	This work
MOR003	MOR002 derivate, inserting *P*_*GAL1*_-*dCas9*-*T*_*AHD1*_-*T*_*SUP4*_-sgRNA1-*P*_*SNR52*_ cassette	This work
MOR004	MOR002 derivate, inserting *P*_*GAL1*_-*dCas9*-*T*_*AHD1*_-*T*_*SUP4*_-sgRNA2-*P*_*SNR52*_ cassette	This work
MOR005	MOR002 derivate, inserting *P*_*GAL1*_-*dCas9*-*T*_*AHD1*_-*T*_*SUP4*_-sgRNA3-*P*_*SNR52*_ cassette	This work
MOR006	MOR002 derivate, Δ*GAL80*:: *P*_*TEF1*_-*ERG1*-*T*_*CYC1*_	This work
MOR007	MOR003 derivate, Δ*GAL80*:: *P*_*TEF1*_-*ERG1*-*T*_*CYC1*_	This work
MOR008	MOR004 derivate, Δ*GAL80*:: *P*_*TEF1*_-*ERG1*-*T*_*CYC1*_	This work
MOR009	MOR005 derivate, Δ*GAL80*:: *P*_*TEF1*_-*ERG1*-*T*_*CYC1*_	This work
MOR010	MOR008 derivate, inserting *P*_*TEF1*_-*ERG1*-*T*_*CYC1*_ cassette	This work
MOR011	MOR010 derivate, inserting *P*_*TEF1*_-*ERG1*-*T*_*CYC1*_ cassette	This work
MOR012	MOR004 derivate, expressing the *CYP87D18* gene via the plasmid pESC-G418	This work
MOR013	MOR010 derivate, expressing the *CYP87D18* gene via the plasmid pESC-G418	This work
MOR014	MOR010 derivate, expressing the *CYP87D18* and *AtCPR1* gene under the control of *P*_*GAL1,10*_ via the plasmid pESC-G418	This work
MOR015	MOR010 derivate, expressing the *CYP87D18* and *AtCPR2* gene under the control of *P*_*GAL1,10*_ via the plasmid pESC-G418	This work
MOR016	MOR010 derivate, expressing the *CYP87D18* and *CsCPR* gene under the control of *P*_*GAL1,10*_ via the plasmid pESC-G418	This work
MOR017	MOR010 derivate, expressing the *CYP87D18* and *SgCPR2* gene under the control of *P*_*GAL1,10*_ via the plasmid pESC-G418	This work
MOR018	MOR010 derivate, expressing the *CYP87D18* and *SrCPR1* gene under the control of *P*_*GAL1,10*_ via the plasmid pESC-G418	This work
FMOR001	CEN-PK2-1C derivate, expressing the plasmid pY13-*P*_*ERG7*_-GFP	This work
FMOR002	CEN-PK2-1C derivate, expressing the plasmid pY13-*P*_*ERG7*_-GFP and pML104-*P*_*GAL1,10*_-dCas9-mCherry-sgRNA-1	This work
FMOR003	CEN-PK2-1C derivate, expressing the plasmid pY13-*P*_*ERG7*_-GFP and pML104-*P*_*GAL1,10*_-dCas9-mCherry-sgRNA-2	This work
FMOR004	CEN-PK2-1C derivate, expressing the plasmid pY13-*P*_*ERG7*_-GFP and pML104-*P*_*GAL1,10*_-dCas9-mCherry-sgRNA-3	This work
LMOR001	CEN-PK2-1C derivate, expressing the plasmid pY15-*P*_*TEF1*_-*SgCDS*-GFP-*T*_*CYC1*_	This work
LMOR002	CEN-PK2-1C derivate, expressing the plasmid pY15- *P*_*TEF1*_-*SgEPH3*-GFP-*T*_*CYC1*_	This work
LMOR003	CEN-PK2-1C derivate, expressing the plasmid pY15- *P*_*TEF1*_-*CYP87D18*-GFP-*T*_*CYC1*_-*P*_*GAP*_-mCherry-Linker-SEC12-*T*_*ADH1*_	This work
LMOR004	CEN-PK2-1C derivate, expressing the plasmid pY15- *P*_*TEF1*_-*AtCPR1*-GFP-*T*_*CYC1*_-*P*_*GAP*_-mCherry-Linker-SEC12-*T*_*ADH1*_	This work

### Yeast Transformation and Strain Construction

*SgCDS*, *SgEPH3*, and *CYP87D18* involved in mogrol synthesis were synthesized and codon-optimized by Genewiz. The CPRs coding *AtCPR1* (GenBank ID: 828554) and *AtCPR2* (GenBank ID: 829144) from *Arabidopsis thaliana* and *SgCPR2* (GenBank ID: AYE89265.1) from *S. grosvenorii* (Zhao et al., 2018) were synthesized and codon-optimized by Genewiz. *SrCPR1* from *Stevia* (Gold et al., 2018) was synthesized and codon-optimized by Genscript (Nanjing, China). CPRs coding *CsCPR* from *Cucumber* were provided by Prof. Sanwen Huang (Chinese Academy of Agricultural Sciences). All genetic modifications used the CRISPR-Cas9-mediated genome editing method and were performed as previously described (Gilbert et al., 2013). Genome integration loci (Reider Apel et al., 2017) and the corresponding single guide RNA (sgRNA) plasmids are listed in Supplementary Table S1. The target fragments were amplified using PCR, and the corresponding plasmid or *S. cerevisiae* genome was used as the template. The primers used in this experiment were synthesized by Genewiz. Overlaps (40–50 bp) of each adjacent fragment were used to implement homologous recombination in yeast. Yeast transformation was performed by the LiAc/SS carrier DNA/PEG method previously described (Gietz and Schiestl, 2007).

### Plasmid Construction

A green fluorescent protein (GFP) was fused with *SgCDS*, *SgEPH3*, *CYP87D18*, and *AtCPR1*, generating plasmids pY15-SgCDS-GFP, pY15-SgEPH3-GFP, pY15-CYP87D18-GFP, and pY15-AtCPR1-GFP, respectively, to determine the localization of heterologous enzymes involved in the biosynthesis pathway of mogrol. Most plant-derived P450 enzymes are located in the endoplasmic reticulum (ER) in *S. cerevisiae* (Arendt et al., 2017). An expression cassette P_GAP_-mCherry-Linker-SCE12-TADH1 was inserted into pY15-CYP87D18-GFP and pY15-AtCPR1-GFP, resulting in plasmids pY15-CYP87D18-GFP-mCherry-SEC12 and pY15-AtCPR1-GFP-mCherry-SEC12, respectively.

*CYP* and *CPR* genes, promoter *P*_*GAL1,10*_, and terminators *T*_*TDH3*_ and *T*_*CYC1*_ were amplified using PCR to construct pESC-G418 series vectors, and the corresponding plasmid or *S. cerevisiae* genome was used as the template. These fragments with a 40-bp homology arm were inserted into multiple cloning sites of pESC-G418 using the Gibson Assembly^®^ Cloning Kit (NEB). Potential sgRNA targets were predicted at http://www.rgenome.net/cas-designer/ to design specific sgRNAs for corresponding targeted genes. All plasmids used in the study are listed in Table 2, and the corresponding primers are listed in Supplementary Table S2.

**TABLE 2:** Plasmids used in the study.

Plasmids	Characteristics	Resource
pESC-Leu	Amp, LEU2, *E. coli*-*S. cerevisiae* shuttle vector	Lab stock
pESC-G418	pESC-LEU2 derivate, Δ*P*_*Leu2*_-LEU2::*P*_*bs*_-KanR-*T*_*tef*_	Lab stock
pML104-dCas9	Amp, URA3, 2μ, *P*_*GAP*_, dCas9, *E.coli*-*S. cerevisiae* shuttle vector	Lab stock
pY13	Amp, HIS3, CEN/ARS, *E. coli*-*S. cerevisiae* shuttle vector	
pY15	Amp, LEU2, CEN/ARS, *E. coli*-*S. cerevisiae* shuttle vector	
pY13-P_ERG7_-GFP	pY13 derivate, Δ*P*_*TEF1*_:: *P*_*ERG7*_-GFP	This work
pML104-dCas9-mCherry	pML104-dCas9 derivate, inserting *T*_*CYC1*_-mCherry-*P*_*GAL1,10*_-dCas9 cassettes in the upstream of dCas9	This work
pML104-dCas9-mCherry-1	pML104-dCas9-mCherry derivate, *P*_*SNR52*_-sgRNA-1 *T*_*SUP4*_	
pML104-dCas9-mCherry-2	pML104-dCas9-mCherry derivate, *P*_*SNR52*_-sgRNA-2 *T*_*SUP4*_	This work
pML104-dCas9-mCherry-3	pML104-dCas9-mCherry derivate, *P*_*SNR52*_-sgRNA-3 *T*_*SUP4*_	This work
pESC-G418-CYP	pESC-G418 derivate, P_GAL1_-CYP-T_TDH3_	This work
pESC-G418-CYP-AtCPR1	pESC-G418 derivate, inserting *T*_*CYC1*_-*AtCPR1*-*PGAL1,10*-*CYP87D18*-*T*_*TDH3*_	This work
pESC-G418-CYP-AtCPR2	pESC-G418 derivate, inserting *T*_*CYC1*_-*AtCPR2*-*PGAL1,10*-*CYP87D18*-*T*_*TDH3*_	This work
pESC-G418-CYP-CsCPR	pESC-G418 derivate, inserting *T*_*CYC1*_-*CsCPR*-*PGAL1,10*-*CYP87D18*-*T*_*TDH3*_	This work
pESC-G418-CYP-SgCPR2	pESC-G418 derivate, inserting *T*_*CYC1*_-Sg*CPR2*-*PGAL1,10*-*CYP87D18*-*T*_*TDH3*_	This work
pESC-G418-CYP-SrCPR1	pESC-G418 derivate, inserting *T*_*CYC1*_-Sr*CPR1*-*PGAL1,10*-*CYP87D18*-*T*_*TDH3*_	This work
pY13-ERG1	pY13 derivate, inserting *P*_*TEF1*_-*ERG1*-*T*_*CYC1*_	This work
pY15- SgCDS-GFP	pY15 derivate, inserting *P*_*TEF1*_-*SgCDS*-GFP-*T*_*CYC1*_	This work
pY15- SgEPH3-GFP	pY15 derivate, inserting *P*_*TEF1*_-*SgEPH3*-GFP-*T*_*CYC1*_	This work
pY15-CYP87D18-GFP-mCherry- SEC12	pY15 derivate, inserting *P*_*TEF1*_-*CYP87D18*-GFP-*T*_*CYC1*_-*P*_*GAP*_-mCherry-Linker-SEC12-*T*_*ADH1*_	This work
pY15-AtCPR1-GFP-mCherry- SEC12	pY15 derivate, inserting *P*_*TEF1*_-*AtCPR1*-GFP-*T*_*CYC1*_-*P*_*GAP*-_mCherry-Linker-SEC12-*T*_*ADH1*_	This work

### Fluorescence Imaging and Analysis

Gene localization of the mogrol biosynthesis pathway was observed in strain CEN-PK2-1C. Strain CEN-PK2-1C was transformed by plasmids pY15-SgCDS-GFP, pY15-SgEPH3-GFP, pY15-CYP87D18-GFP-mCherry-SEC12, and pY15-AtCPR1-GFP-mCherry-SEC12, thereby yielding strains LMOR001, LMOR002, LMOR003 and LMOR004. These strains were streaked onto an SD-Leu plate and cultured at 30°C for 2 days. Later, a single colony was picked into a 2 ml SD-Leu medium, cultured at 30°C and 220 rpm for 24 h, and washed twice with 1× phosphate-buffered saline (0.8% NaCl, 0.02% KCl, 0.144% Na_2_HPO_4_, and 0.024% KH_2_PO_4_, pH 7.4). Subsequently, 2-μL preparations were directly plated on slides. Strain LMOR001 culture was stained with Nile red solution in acetone (1:10, v/v; 1 mg/ml; Solarbio) and incubated for 60 min in the dark at 25°C to further confirm the specific distribution of *SgCDS*.

Fluorescence imaging was performed on a Nikon C-HGF microscope with a ×100 oil immersion objective. GFP fluorescence (excitation, 490 nm; emission, 530 nm), mCherry fluorescence (excitation, 580 nm; emission, 615 nm), and Nile red fluorescence (excitation, 561 nm; emission, 615 nm) were detected by microscopy. Image analysis was carried out on the Leica TCS SP8 software package and ImageJ (NIH).

### Fluorescence Detection

The endogenous promoter of *ERG7* and the *gfp* gene were inserted into plasmid pY13 to construct plasmid pY13-P_ERG7_-GFP. Strain CEN-PK2-1C was transformed by plasmid pY13-P_ERG7_-GFP to yield strain FMOR001. The mCherry protein shared the bidirectional promoter *P*_*GAL1,10*_ with the dcas9 protein on plasmid pML104-dCas9. The corresponding sgRNA was integrated into plasmid pML104-dCas9 to construct plasmid pML104-dCas9-mCherry-1/2/3. Strain CEN-PK2-1C was transformed by plasmids pY13-P_ERG7_-GFP and pML104-dCas9, thereby yielding strains FMOR002, FMOR003, and FMOR004, respectively. These strains were streaked onto an SD-Ura-His (FMOR002, FMOR003 and FMOR004) or SD-His (FMOR001) plate and cultured at 30°C for 2 days. Later, a single colony was picked into a 2 ml SD-Ura-His or SD-His medium and cultured at 30°C and 220 rpm for 24 h. The 2% (v/v) seed cultures were inoculated into a 200 μL SD-Ura-His or SD-His medium in a 96-well fluorescent plate and cultured at 30°C for 72 h. Strains with plasmids pML104-dCas9 and pY13-P_ERG7_-GFP were cultivated in the SD-Ura-His medium, and the strain with pY13-P_ERG7_-GFP was cultivated in the SD-His medium. All strains containing plasmid pML104-dcas9 needed 2%, 4%, 6%, 8%, 10%, and 20% galactose at 0 h. GFP fluorescence (excitation, 490 nm; emission, 530 nm), mCherry fluorescence (excitation, 580 nm; emission, 615 nm), and OD600 of strains were detected using a Cytation microplate reader (BioTek). Eq. 1 was used to calculate the repression fold of GFP fluorescence (*θ*) by different sgRNAs.

$$\theta =\frac{\left[GFP/OD\right]1-\left[GFP/OD\right]0}{\left[GFP/OD\right]x-\left[GFP/OD\right]0}-1$$ (1)

where (GFP/OD)_x_ is the relative fluorescence intensity of GFP in strain FMOR002, FMOR003 or FMOR004; (GFP/OD)_0_ is the relative fluorescence intensity of GFP in strain MOR001 (without GFP); and (GFP/OD)_1_ is the relative fluorescence intensity of GFP in strain FMOR001.

### Heterologous Expression in Recombinant Yeast and Analysis

The strains were streaked onto a YPD solid plate and cultured at 30°C for 2 days. A single colony was picked into a 2 ml YPD medium at 30°C and 220 rpm for 24 h. The 1% (v/v) cultures were inoculated into a 250 ml flask containing 50 ml YPD medium. Strains containing plasmid pESC-G418 needed 200 mg/L G418 to add to the YPD medium. All flask fermentation results represented the mean ± standard deviation (SD) of three independent experiments.

A 1 ml fermentation culture was collected and broken by a high-pressure homogenizer (FastPrep-24 5G, United States ) and extracted twice with ethyl acetate to measure the mogrol and squalene concentrations. For squalene analysis (Paramasivan and Mutturi, 2017), the samples were filtered using a 0.45 μm organic phase filtration membrane and injected into a high-performance liquid chromatography instrument (Agilent 1260 series). The column was a C18 ODS column (5 μm, 250 × 4.6 mm; Thermo Fisher Scientific), and the column temperature was 40°C. The pump flow rate was 1.6 ml/min, and the mobile phase was 100% acetonitrile. The injection volume was fixed to 10 μL. For mogrol analysis, the samples extracted with ethyl acetate were resuspended in 100 μL methanol after evaporation. Mogrol was quantified using liquid chromatography mass spectrometry (LC–MS) according to Itkin et al. (2016).

## Results

### Construction of the *de novo* Biosynthetic Pathway of Mogrol in *S. Cerevisiae*

According to the mogrol synthetic pathway in plants, the initial step in mogrol biosynthesis is the epoxidation of 2, 3-oxidosqualene to form 2, 3; 22, 23-diepoxysqualene (the triterpenoid skeleton of mogrol). Then, 2, 3; 22, 23-diepoxysqualene is cycled into 24, 25-epoxycucurbiadienol by SgCDS. SgEPH3 catalyzes the unique hydroxylation of 24, 25-epoxycucurbiadienol to form *trans*-24, 25-dihydroxycucurbitadienol. Finally, CYP87D18 adds an oxygen atom to position C11 of 24, 25-dihydroxycucurbitadienol to form mogrol (Figure 1).

**FIGURE 2:**
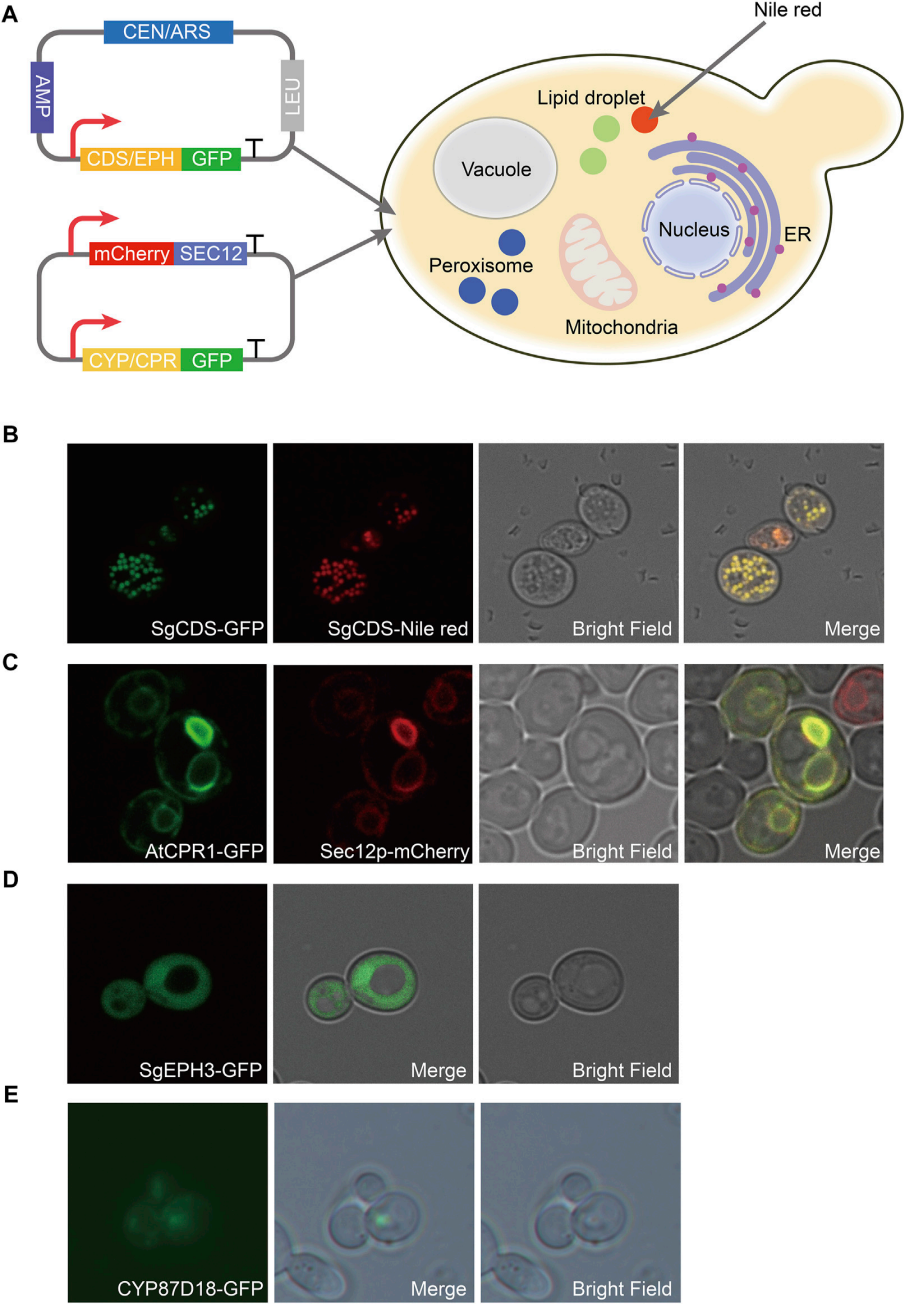
**Distribution of enzymes involved in the synthesis pathway of mogrol. (A)** Construction of plasmids pY15-GFP and pY15-GFP-mCherry. SEC12 was proven to be an ER-localized protein. GFP protein was used to visualize the localization of the target protein. mCherry protein was used to specifically mark the ER, and Nile red was used to show intracellular lipophilic regions **(B–E)** Distribution analysis of *SgCDS*, *SgEPH*, *CYP87D18*, and *AtCPR1* by LSCM, respectively.

Therefore, to construct the *de novo* biosynthetic pathway of mogrol in *S. cerevisiae*, *SgCDS*, *SgEPH3*, and *CYP87D18* from *S. grosvenorii* and the CPR gene *AtCPR1* from *A. thaliana* were integrated into the chromosome of strain Y4 (a high-yield strain of squalene constructed previously), yielding strain MOR001. The expression of these genes was under the control of the galactose-inducible promoter *P*_*GAL1,10*_. Synthetic genes were first employed as templates and then the genes were amplified to investigate the expression and distribution of SgCDS, SgEPH3, CPY87D18, and AtCPR1. Genes were integrated into plasmids, and the *gfp* gene was fused downstream of them (Figure 2A). These plasmids were transformed into strain MOR001, thereby yielding strains LMOR001, LMOR002, LMOR003 and LMOR004. Comparing the fluorescent images of SgCDS to the bright-field images, the green fluorescent spots showed a point-like distribution. These apparent traits showed that these spots are like lipid drops (LDs), the primary storage sites for neutral lipids in cells (Hammer and Avalos, 2017). Then, cells were stained with Nile red, a dye that can directly stain LDs (Liu et al., 2020). The spots in Nile red images exactly overlapped with the green fluorescent spots. It was concluded that SgCDS was specifically localized in LDs (Figure 2B), which was similar to the cycloartenol synthase from *Arabidopsis thaliana* (Milla et al., 2002) and α-amyrin synthase from *Malus × domestica* (Yu et al., 2020). According to the merged results of the colocalization of AtCPR1 and ER, the localization of GFP fluorescence was consistent with sec12p, suggesting that AtCPR1 was an ER-localized protein in *S. cerevisiae* (Figure 2C). Besides, laser scanning confocal microscopy (LSCM) analysis revealed that the GFP fluorescence of SgEPH3 filled the entire cytoplasm of *S. cerevisiae*, showing that SgEPH3 was located in the cytoplasm (Figure 2D). Furthermore, the GFP fluorescence of CYP87D18 was weak, indicating that the enzyme activity of CYP87D18 was a bottleneck in the biosynthesis pathway of mogrol (Figure 2E). These results proved that heterologous SgCDS, SgEPH3, CPY87D18 and AtCPR1 genes were successfully expressed in strain MOR001.

**FIGURE 3:**
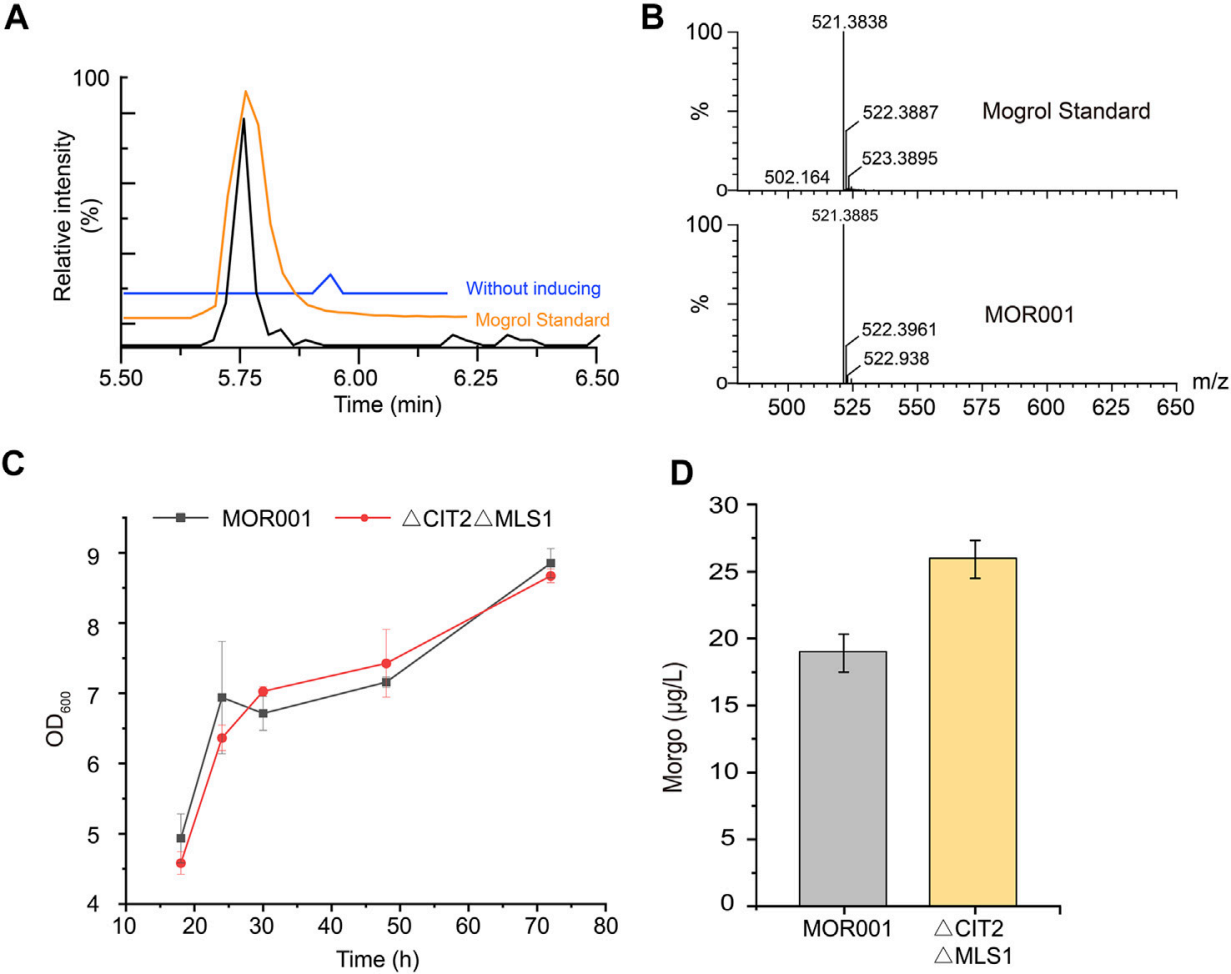
**LC–MS analysis of mogrol produced by strain MOR001. (A,B)** Chromatogram analysis **(A)** and mass spectrogram analysis **(B)** of MOR001 and mogrol standard. The blue line in **(A)** represents strain MOR001 in the YPD medium without induction with galactose **(C,D)** Cell growth curves **(C)** and mogrol titer **(D)** in engineered strain MOR002, in which *MLS1* and *CIT2* genes were knocked out. Error bars represent the SD of biological triplicates (*n* = 3).

After strain MOR001 cultivation, a peak with a mass-to-charge ratio (*m/z*) value of 521.389 was detected using LC–MS in strain MOR001, in accordance with the *m/z* values of mogrol standard (Figures 3A,B). Mogrol could only be detected when the inducer galactose was added to the medium. These results demonstrated that the *de novo* biosynthetic pathway of mogrol was successfully constructed in strain MOR001. However, the mogrol titer in strain MOR001 was only 19.1 ng/L. The metabolic network was divided into three modules, including the squalene, 2, 3; 22, 23-diepoxysqualene, and mogrol synthesis modules, to synergistically regulate the metabolic network of mogrol (Figure 1). For the squalene synthesis module, the precursor acetyl-CoA supply was enhanced to facilitate squalene synthesis. In the cytoplasm, CIT2 and MLS1 involved in the glyoxylate pathway consumed intracellular acetyl-CoA. Therefore, *CIT2* and *MLS1* were knocked out to reduce the consumption of the precursor acetyl-CoA using the CRISPR–Cas9 system, generating strain MOR002. However, the mogrol titer in strain MOR002 only reached 25.9 ng/L, which was 1.3-fold higher than that of MOR001 (Figure 3D). These results indicated that the intracellular content of acetyl-CoA and squalene was sufficient to supply mogrol synthesis, which may be due to the high content of precursor squalene in the original strain Y4.

### Inhibition of the Sterol Synthesis Pathway Using the CRISPRi System

In the 2, 3; 22, 23-diepoxysqualene synthesis module, Erg7p (lanosterol synthase) catalyzed the cyclization of (3*S*)-2, 3-oxidosqualene to lanosterol and further flowed into the synthesis pathway of ergosterol, one of the main components of the plasma membrane (Nes, 2011; Jordá and Puig, 2020). In general, 2,3-oxidosqualene prefers to be cyclized by ERG7 rather than oxidized into 2, 3; 22, 23-diepoxysqualene (Corey and Gross, 1967; Shan et al., 2005). Therefore, knocking out or inhibiting the activity of ERG7 is crucial for 2, 3; 22, 23-diepoxysqualene synthesis. However, directly blocking the metabolic flux to ergosterol would affect cell growth and require additional supplementation of ergosterol and Tween 80 in the medium to maintain cell growth, which was not cost-saving and conducive to industrial development (Itkin et al., 2016; Qiao et al., 2019).

**FIGURE 4:**
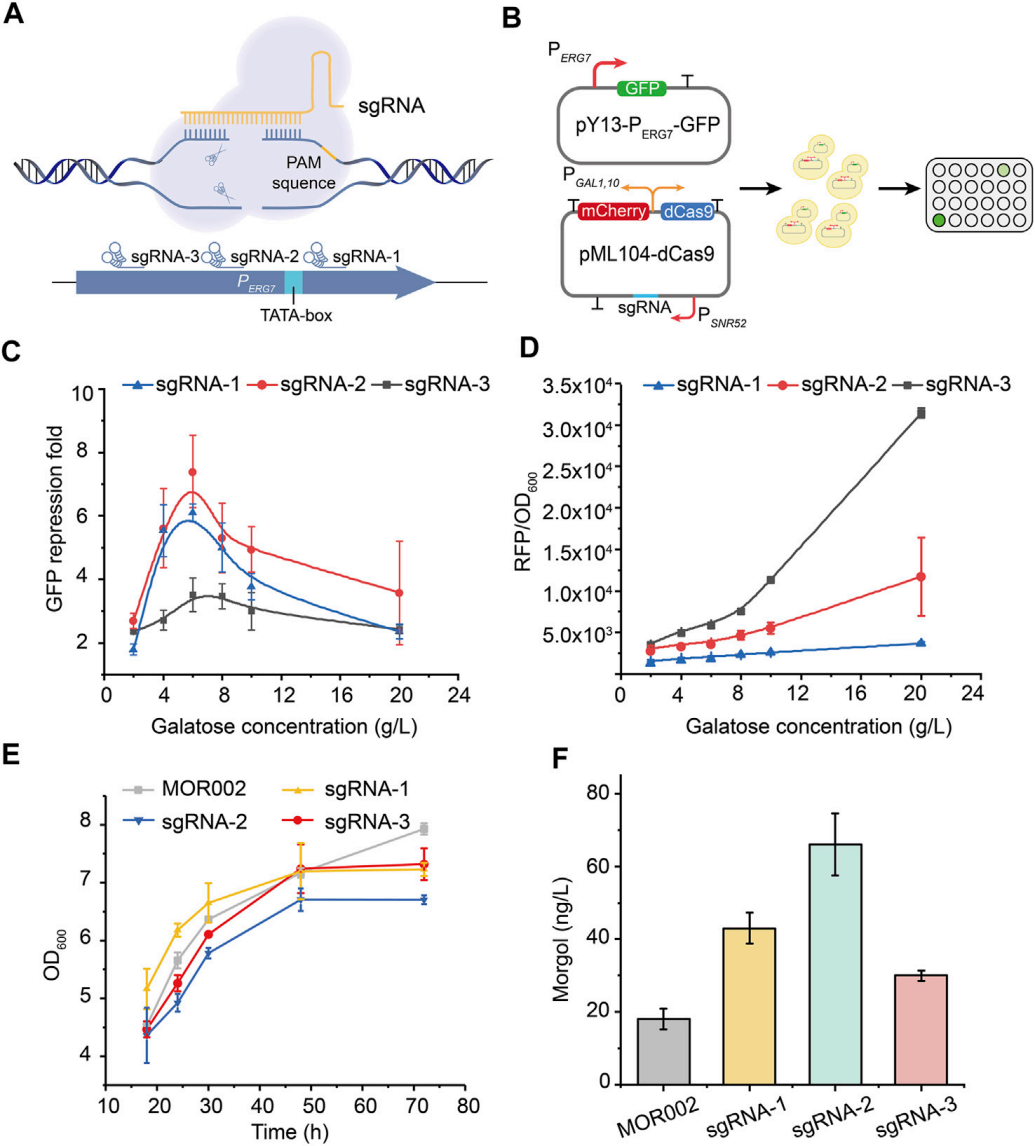
**Repression of the lanosterol biosynthesis pathway by the CRISPRi system. (A)** CRISPRi was employed to repress gene expression by an inactive Cas9 protein (dCas9), which can bind to a specific sequence by sgRNA and block the elongation of RNA polymerase. sgRNAs were chosen from the endogenous *ERG7* promoter. **(B)** Construction of plasmids that characterize the feasibility of the CRISPRi system in yeast and the verification of fluorescence in a 96-well plate. **(C)** GFP fluorescence repression fold of different sgRNAs of promoter *P*_*ERG7*_. **(D)** mCherry fluorescence intensity of dCas9 protein. **(E)** Cell growth curves of engineered strains in a 250-ml flask culture in a YPG medium **(F)** Mogrol production in engineered strains at 96 h. In **(E)** and **(F)**, sgRNA-1 represented strain MOR003, sgRNA-2 represented strain MOR004, sgRNA-3 represented strain MOR005. Error bars represent the SD of biological triplicates (*n* = 3).

The CRISPRi system is widely used to inhibit competing pathways in metabolic networks of various products (Gilbert et al., 2013; Jensen et al., 2017; Wu et al., 2018). Therefore, the CRISPRi system was used to inhibit *ERG7* expression, thereby redirecting the metabolic flux toward mogrol biosynthesis, enabling the decoupling of cell growth and product synthesis. First, the inhibitory targets of the CRISPRi system on the promoter of *ERG7* (*P*_*ERG7*_) and their corresponding inhibitory effects were screened using the *gfp* reporter gene (Figure 4A). Control plasmid pY13-P_ERG7_-GFP containing the reporter gene *gfp* was constructed to characterize the transcription strength of promoter *P*_*ERG7*_. Three sgRNAs targeting different positions of the promoter *P*_*ERG7*_ were inserted into plasmid pML104-dCas9-mCherry, which contained a tandem expression cassette dCas9-mCherry under the control of galactose-inducible promoter *P*_*GAL1,10*_, thereby obtaining plasmids pML104-dCas9-mCherry-1/2/3 (Figure 4B). The dCas9 expression level in plasmid pML104-dCas9-mCherry-1/2/3 was characterized according to the fluorescence intensity of mCherry. The control plasmid pY13-P_ERG7_-GFP was transformed into the wild-type strain CEN-PK2-1C, yielding strain FMOR001. Plasmids pY13-P_ERG7_-GFP and pML104-dCas9-mCherry-1/2/3 were cotransformed into the wild-type strain CEN-PK2-1C, thereby yielding strain FMOR002, FMOR003, and FMOR004 (Figure 4B). Finally, the fluorescence intensity of GFP and mCherry in strains FMOR001, FMOR002, FMOR003, and FMOR004 was measured. The results showed that when the inducer galactose concentration was 6 g/L, the repression fold of GFP fluorescence (*θ*) in strains FMOR002, FMOR003 and FMOR004 were 6.1-, 7.4-, and 3.5-fold, respectively (Figure 4C, Supplementary Figure S1). When the galactose concentration was >6 g/L, the repression fold of GFP fluorescence (*θ*) by different sgRNAs was gradually decreased (Figure 4C). In addition, the expression level of dCas9 in strains FMOR004 was higher than FMOR002 and FMOR003, but the repression fold of GFP fluorescence (*θ*) in strains FMOR004 were lowest (Figure 4D). This result indicating that sgRNA rather than the dCas9 protein was the most essential for the efficiency of the CRISPRi system.

Subsequently, dcas9-sgRNA expression cassettes in plasmids pML104-dCas9-mCherry-1/2/3 were cloned and integrated into the genome of strain MOR002 to repress *ERG7* expression, thereby yielding strains MOR003, MOR004, and MOR005, respectively. The growth of strains MOR003, MOR004, and MOR005 was similar to strain MOR002 (Figure 4E), demonstrating that the CRISPRi system could inhibit *ERG7* expression without affecting cell growth. Meanwhile, the mogrol titer in strains MOR003, MOR004, and MOR005 reached 43, 66, and 30 ng/L, increased by 53%, 135%, and 7%, respectively, compared to that of MOR002 (Figure 4F), consistent with the GFP fluorescence repression fold of different sgRNAs.

### Improving Mogrol Production by Enhancing Copies of *ERG1*

**FIGURE 5:**
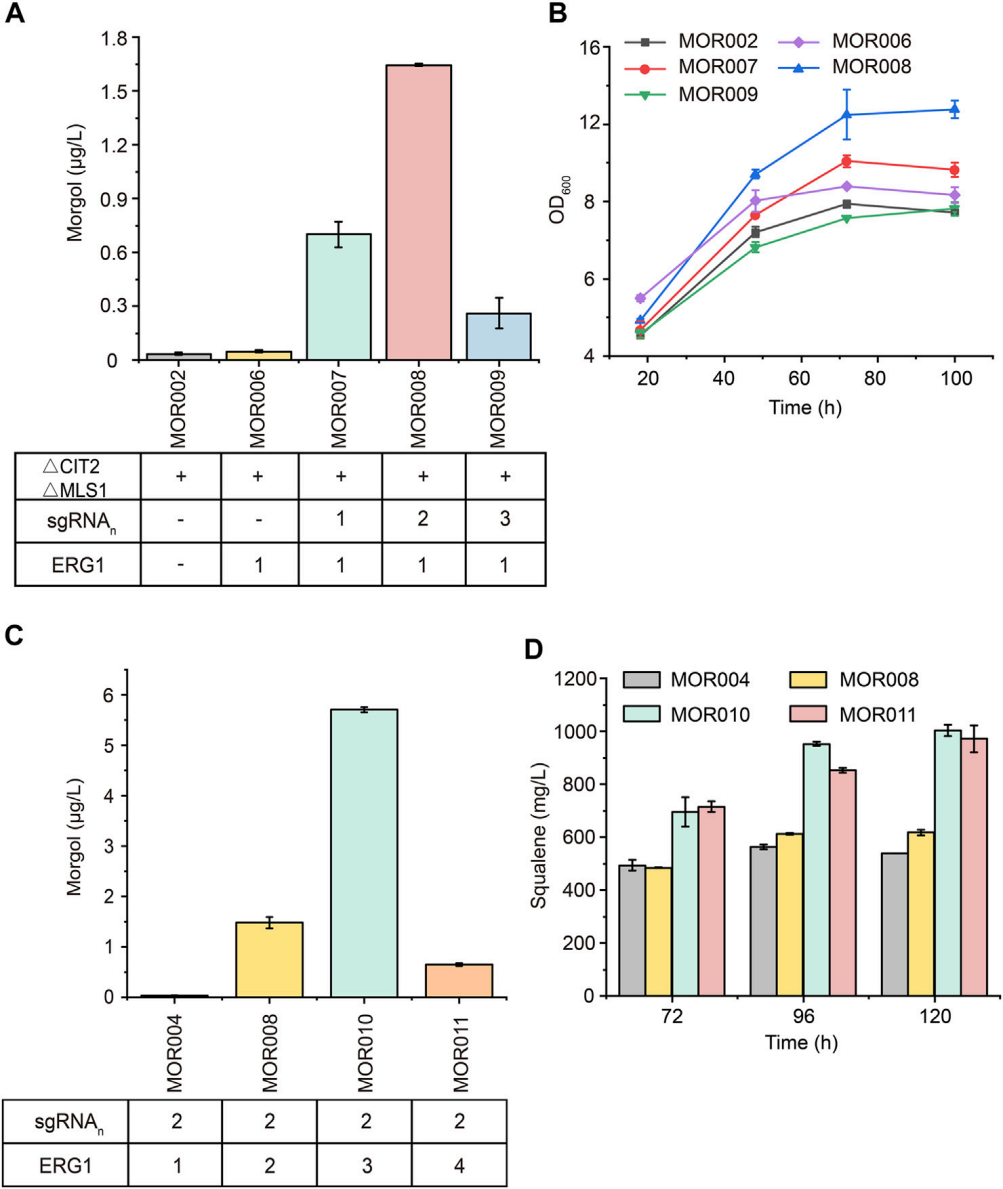
**Effects of multicopy expression of *ERG1* on mogrol. (A)** Mogrol production in MOR007 to MOR009 compared to MOR002 and MOR006 **(B)** Cell growth curves of engineered strains that overexpressed *ERG1* in YPD medium. **(C,D)** Mogrol **(C)** and squalene **(D)** production after multicopy genome integration of *ERG1*. Error bars represent the SD of biological triplicates (*n* = 3).

To further improve mogrol production, the metabolic flux of the mogrol synthesis module was engineered. First, the precursor 2, 3-oxidosqualene flow was “pulled” into the mogrol synthesis module rather than cell growth by overexpressing *ERG1* (squalene epoxidase), which catalyzed squalene to 2, 3-oxidosqualene and 2, 3; 22, 23-diepoxysqualene. Another copy of *ERG1* was integrated into the genome of strain MOR002, and *GAL80* (a transcriptional factor) (Pilauri et al., 2005) was simultaneously knocked out, yielding strain MOR006. LC–MS results showed that the mogrol titer in strain MOR006 reached 48 ng/L, which showed a further 240% increase than that of MOR002 (Figure 5A). When another copy of *ERG1* was integrated into the genome of strains MOR003, MOR004 and MOR005, the mogrol titer in the resulting strains MOR007, MOR008 and MOR009 were 13.9-, 33-, and 4.8-fold as high as that of strain MOR006, reached 670, 1600, and 230 ng/L, respectively (Figure 5A). These results indicated that only inhibiting the synthetic pathway of lanosterol and synergistically “pulling” the metabolic flux flow into the 2, 3; 22, 23-diepoxysqualene could maximize mogrol production. Subsequently, the copy number of *ERG1* in strain MOR008 was further increased to improve mogrol production. When the copy number of *ERG1* increased to three copies (MOR010), the mogrol titer was further increased to 5.67 μg/L. When the copy number of *ERG1* reached four copies (MOR011), the mogrol titer decreased to 0.68 μg/L (Figure 5C). Finally, the titer of the intermediate metabolite squalene was also measured in strains MOR004, MOR008, MOR010, and MOR011. The results showed that the squalene titer in strains MOR008, MOR010 and MOR011 at 120 h reached 617, 1002.9, and 971.4 mg/L, which was 1.1-, 1.86- and 1.81-fold higher respectively, compared to that of the strain MOR004 (Figure 5D). The biomass of MOR008, MOR010 and MOR011 was significantly improved compared to MOR004, which may be due to squalene being an essential precursor for cells to synthesize the plasma membrane (Figure 5B). These results demonstrated that *ERG1* increased mogrol production by “pulling” the metabolic flux toward the biosynthesis pathway of mogrol.

### Optimization of the Expression and Reduction System of P450 Enzymes

Most plant-derived P450 enzymes (CYP) are located in the ER and are often considered as the rate-limiting enzymes in the biosynthetic pathway (Hausjell et al., 2018). They rely on CPRs to obtain electrons from NAD(P)H for subsequent catalytic reactions (Sadeghi and Gilardi, 2013). Many studies showed that different sources of CPRs may affect CYP activity, and the compatibility of native CPRs with CYPs in heterologous hosts is often not optimal (Theron et al., 2019). Hence, screening CPRs from different sources is an effective strategy to increase the activity of P450 enzymes.

**FIGURE 6:**
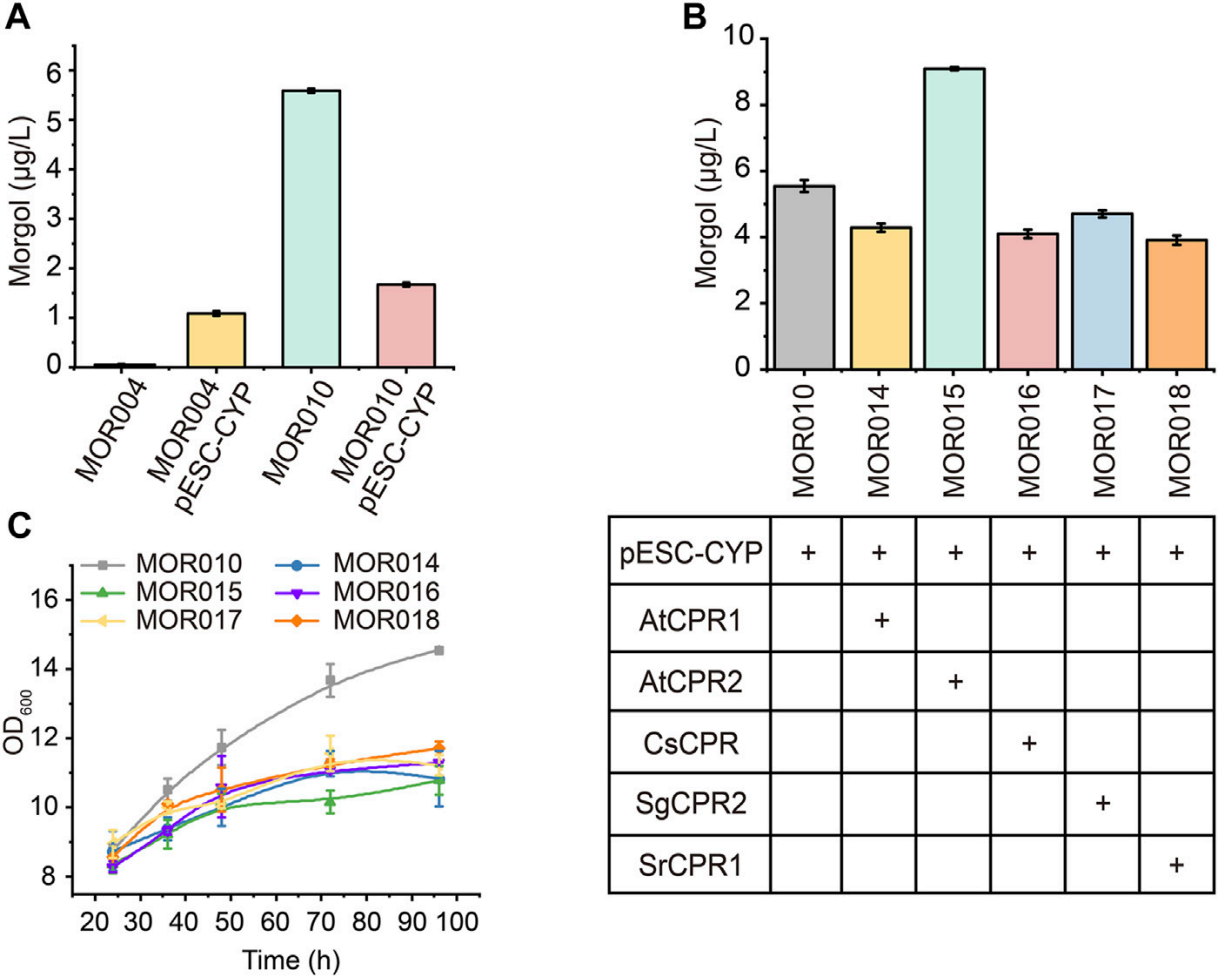
**Combined optimization of CYPs-CPRs pairing to improve mogrol production. (A)** Mogrol production by overexpressing *CYP87D18* using a high-copy plasmid. **(B,C)** Mogrol production by optimizing CYPs-CPRs pairing **(B)** and cell growth curves of engineered strains **(C)** in a 250-ml flask. Error bars represent the SD of biological triplicates (*n* = 3).

Therefore, *CYP87D1*8 was first inserted into a high-copy plasmid pESC-G418 to test whether increasing its expression would improve mogrol production, yielding plasmid pESC-G418-CYP. Plasmid pESC-G418-CYP was transformed to MOR004 and MOR010 to construct strains MOR012 and MOR013, respectively. Compared to MOR004, *CYP87D18* expression on a multicopy plasmid significantly increased mogrol production (1.05 μg/L; Figure 6A). This result revealed that increasing P450 enzyme expression was beneficial to mogrol synthesis, whereas it decreased in strain MOR013 (1.64 μg/L) compared to strain MOR010 (5.67 μg/L).

Subsequently, *CYP87D18* was coexpressed in combination with five different CPRs in plasmid pESC-G418, including *AtCPR1* and *AtCPR2* from *A. thaliana*, *SgCPR2* from *S. grosvenorii* (Zhao et al., 2018), *CsCPR* from *Cucumber*, and *SrCPR1* from *Stevia* (Gold et al., 2018). The resulting plasmids were transformed into strain MOR010, thereby obtaining strains MOR014, MOR015, MOR016, MOR017, and MOR018, respectively. Fermentation data showed that the mogrol titer in strain MOR015 reached 9.1 μg/L, which was 1.6-fold higher than that of the strain MOR010. In contrast, the mogrol titer in strains MOR014 MOR016, MOR017 and MOR018 was lower than that of MOR010 (Figure 6B). These results demonstrated that the selection of appropriate CPR is crucial for the catalytic efficiency of CYPs in heterologous hosts. Additionally, the biomass of these strains was only 70% of MOR010 (Figure 6C), which may be due to the additional burden on the organism caused by that the overexpression of the second recombinant protein (Hausjell et al., 2018).

## Discussion

As the aglycone of mogrosides, mogrol has a significant effect on reducing blood glucose and blood lipid. However, more studies focused on the biological properties of mogrol than on its biosynthesis. Additionally, the biosynthesis pathways for mogrol from other precursors have not been explored (Li et al., 2019; Qiao et al., 2019). *S. cerevisiae* is an ideal host for the heterologous synthesis of plant-derived natural products due to its ability to express P450 enzymes located in the ER. Therefore, this study aimed to construct a yeast cell factory for efficient mogrol production. First, a *de novo* synthesis pathway for mogrol was successfully constructed in *S. cerevisiae*. Subsequently, to improve the biosynthesis efficiency of the chassis cell, the metabolic network was divided into three modules. For the squalene synthesis module, the central metabolite acetyl-CoA flow was promoted into the squalene synthesis pathway by inhibiting its flow into the glyoxylate cycle. Surprisingly, in this study, directly knocking out *CIT2* and *MLS1* involved in the glyoxylate cycle would not affect cell growth (Figure 3C). However, Liu et al. (2019) knocked out *CIT2* and *MLS1* in a chassis cell of β-amyrin, resulting in cell growth inhibition. This may be caused by changes in the metabolic network of cells due to the introduction of different heterologous pathways. In addition, our results indicated that the content of the precursor squalene was sufficient for the synthesis of mogrol. Compared to the squalene synthesis module, the low metabolic fluxes of the 2, 3; 22, 23-diepoxysqualene synthesis and mogrol synthesis module may be the major rate-limiting steps for the synthesis of mogrol. Therefore, we subsequently enhanced the metabolic fluxes of 2, 3; 22, 23-diepoxysqualene synthesis module and mogrol synthesis module.

In 2, 3; 22, 23-diepoxysqualene synthesis module, ERG1 was the only enzyme involved. ERG1 is a key rate-limiting enzyme in the sterol biosynthetic pathway. When the intracellular concentration of lanosterol is high, its activity will be feedback-inhibited, thereby maintaining the homeostasis of sterols. Therefore, reducing the intracellular sterol content is crucial for maintaining the catalytic activity of ERG1. Inhibiting *ERG7* (related to sterol synthesis pathway) expression will promote ERG1 to catalyze the synthesis of 2, 3; 22, 23-diepoxysqualene from squalene (Xu et al., 2004; Zhao et al., 2019). The traditional methods of inhibiting ERG7 expression were mainly by replacing the native *P*_*ERG7*_ promoter with an inducible promoter, such as methionine-inhibited promoter *MET3* (Baadhe et al., 2013) and a copper-inhibited promoter *CTR3* (Paddon et al., 2013; Rodriguez et al., 2016). However, adding methionine and copper ions will increase production costs, and copper ions are toxic to yeast. Therefore, the CRISPR-dCas9 system was employed to inhibit *ERG7* expression. Three sgRNAs that inhibited *ERG7* expression in different strengths were screened. sgRNA-2 had the best inhibitory effect on *ERG7* expression, probably because its target was closest to the TATA-box of *P*_*ERG7*_ (Figure 3). Moreover, the mogrol titer gradually increased with the inhibited effect of sgRNA. These results confirmed that reducing the metabolic flux of the sterol pathway is an effective way to increase mogrol production. More importantly, compared with other modules, the yield of mogrol was improved most obviously by adjusting this module. Furthermore, although ERG1 could catalyze the synthesis of 2, 3; 22, 23-diepoxysqualene from squalene, its activity may be low due to 2, 3; 22, 23-diepoxysqualene is not a known precursor for a metabolite in *S. cerevisiae*. Therefore, the activity of ERG1 is likely a major bottleneck for mogrol biosynthesis in *S. cerevisiae*. In future work, screening for a more efficient squalene epoxidase, or using enzyme engineering techniques to enhance the catalytic activity of ERG1, is necessary to increase the production of mogrol.

In mogrol synthesis module, the critical engineering step for mogrol overproduction was the low catalytic efficiency of P450 catalytic system. P450 enzymes are generally considered to be the rate-limiting step for triterpene synthesis (Li et al., 2021). In this study, *CYP87D18* expression in MOR001 was weak. Therefore, for the mogrol synthesis module, *CYP87D18* was expressed on the high-copy plasmid in strain MOR004, resulting in a 12-fold mogrol production. This result demonstrated that *CYP87D18* might be a rate-limiting factor for improving the mogrol titer. While overexpressing *CYP87D18* in strain MOR010, mogrol production decreased. This may be due to an imbalance of the cofactor NADPH in the cytoplasm, as Erg9p (Paramasivan and Mutturi, 2017), Erg1p (Ruckenstuhl et al., 2007) and P450 enzymes will consume NADPH for catalytic reactions. If the expression of the P450 enzyme and Erg1p increases, it may lead to an insufficient supply of intracellular NADPH. Most eukaryotic P450s are complex membrane proteins and require their redox partner CPRs for electron transfer (Jiang et al., 2021). Besides, a suitable CYP-CPR pairing is usually beneficial for the catalytic efficiency of P450 enzymes (Chen et al., 2021). The electron transfer efficiency between them will further affect the subsequent catalytic ability of P450 enzymes. Theron et al. (2019) found that a coexpressed CPR from *Ustilago maydis* (UmCPR) with CYP53B1 resulted in a six-to seven-fold p-hydroxybenzoic acid volumetric yield than that of the endogenous CPR. Additionally, Zhu et al. (2018) introduced a novel CPR reduction system to the metabolic network of 11-oxo-β-amyrin and glycyrrhetinic acid (GA), and the 11-oxo-β-amyrin and GA titer increased by 1422- and 946.5-fold, which reached 108.1 and 18.9 mg/L, respectively. CPRs from different sources have different catalytic efficiencies for P450 enzymes, which will further affect the production synthesis. In this study, by selecting CPR from different sources, *AtCPR2* from *A. thaliana* had the highest electron transfer efficiency among the candidate CPRs, and the mogrol titer was 1.6-fold higher compared to that of MOR010. Meanwhile, we found that expressing CYP and CPRs through a high-copy plasmid resulted in a reduction of biomass. When the reaction ratio of CYP and CPR was not optimal, the excessive electron transport might lead to the release of reactive oxygen species (ROS) and cause cell damage (Zhao et al., 2017; Lin et al., 2022).

Though we significantly improved the mogrol titer by optimizing the expression of key genes of the mogrol metabolic network and improving the catalytic activity of P450 enzyme, the mogrol titer was still very low. We speculated that the activity of squalene epoxidase ERG1 and P450 enzyme might be the main rate-limiting steps that limited the synthesis of mogrol. Generally, squalene epoxidase only has the function of cyclizing squalene to form 2, 3-oxidosqualene. However, Itkin et al. (2016) found that squalene epoxidase derived from *S. grosvenorii* could further catalyze the synthesis of 2, 3; 22, 23-diepoxysqualene from 2, 3-oxidosqualene. ERG1 could perform the same function in yeast, but this catalytic step may be a side reaction because 2, 3; 22, 23-diepoxysqualene is not a known precursor for a metabolite in *S. cerevisiae*. Moreover, the enzymatic activity of P450 enzyme is the rate-limiting step that restricts the efficient synthesis of most terpenoids in *S. cerevisiae* (Hausjell et al., 2018; Jiang et al., 2021). Herein we improved the catalytic activity of P450 enzymes by paring it with a most suitable CPR, and we will carry out directed evolution of P450 enzyme to further improve the catalytic performance in the future. The titers of various hydrophobic terpenoids were improved through compartmentalizing their metabolite pathways into LDs, such as lycopene (Ma et al., 2019), α-amyrin (Yu et al., 2020), and ginsenosides (Shi et al., 2021). Therefore, compartmentalizing ERG1 and the enzymes involved in mogrol synthesis module into LDs may be an effective approach to increase the mogrol production. In summary, we achieved the bioproduction of mogrol by the engineered yeast cell factories, and the used metabolic engineering strategies may be useful for the bioproduction of the other natural products in yeast.

## Data Availability

The original contributions presented in the study are included in the article/ **Supplementary Material** , further inquiries can be directed to the corresponding author.
