# Supplementary material for: Construction and Optimization of the de novo Biosynthesis Pathway of Mogrol in Saccharomyces Cerevisiae
Source: Front Bioeng Biotechnol. 2022 May 27;10:919526. doi: 10.3389/fbioe.2022.919526 (PMC9197265; doi:10.3389/fbioe.2022.919526)
Supplement: Supplementary file 1 [file DataSheet1.DOCX]

**Supplemental Information**

Table S1 SgRNA sequence

| **Name** | **Sequence** |
| --- | --- |
| P_ERG7_-SgRNA-1 | TCTTTGTGGGCGACGATTATTGG |
| P_ERG7_-SgRNA-2 | ATGATTGCAAATTAAAATAGGGG |
| P_ERG7_-SgRNA-3 | GCATGATTAGCAGAGACATATGG |
| 1622b | TAAAGCCACCACATCGCAAA |
| 208a | GTCCGCTAAACAAAAGATCT |
| 106a | ATACGGTCAGGGTAGCGCCC |
| 1021b | CCTCTGTGTGGTGGTAATTG |
| 308a | CACTTGTCAAACAGAATATA |
| ΔCIT2 | GTTAGTTTCATCAATATACGAGG |
| ΔMLS1 | AAATGGTGAGATTACTACCGAGG |
| ΔGAL80 | TAATATCAGAGCATCCATCAAGG |

Table S2 List of primers used in this study

| **Primer name** | **Sequence (5′-3′)** |
| --- | --- |
| **For plasmids construction** |  |
| P_ERG7_-F | ccttacccatcatctgttttgtactttctttgtgggc |
| P_ERG7_-R | gctggagctctccggatattgtgttaaaatttcaagt |
| GFP-F | ataactaattacatgattatttgtacagttcatccatgccatg |
| GFP-R | aaaacagatgatgggtaagggagaagaacttttc |
| pY13-GFP-F | gtacaaataatcatgtaattagttatgtcacgcttacatt |
| pY13-GFP-R | aatatccggagagctccagcttttgttccc |
| mCherry-F | agatttaaagtaaattcactttacttgtacagctcgtccatg |
| mCherry-R | tttttgaaaattcaatataaatggccatcatcaaggagttcat |
| T_TDH3_-dcas9-F | tactagtcagttcgagtttaaagggaaagatatgagctatacagc |
| T_TDH3_--mCherry-R | agtgaatttactttaaatcttgcatttaaataaat |
| pML104-dCas9-F | taaactcgaactgactagtagactgaattcg |
| pML104-dCas9-R | agatctgatatggatcgaattagatctcg |
| P_GAL1,10_-dCas9-F | aattcgatccatatcagatcttatagttttttctccttgacgttaaagtataga |
| P_GAL1,10_-dCas9-R | ttatattgaattttcaaaaattcttactttttttttggatggacgc |
| sgRNA1-F | tctttgtgggcgacgattatgttttagagctagaaatagcaagttaaaataaggct |
| sgRNA1-R | ataatcgtcgcccacaaagagatcatttatctttcactgcggagaag |
| sgRNA2-F | ctattttaatttgcaatcatgatcatttatctttcactgcggagaagt |
| sgRNA2-R | atgattgcaaattaaaataggttttagagctagaaatagcaagttaaaataagg |
| sgRNA3-F | gcatgattagcagagacatagttttagagctagaaatagcaagttaaaataagg |
| sgRNA3-R | tatgtctctgctaatcatgcgatcatttatctttcactgcggagaagt |
| CYP87D18-F | atactttaacgtcaaggagaaaaaactataatgtggactgttgttttgggtttg |
| CYP87D18-R | ccttttcggttagagcggatttattcttttggagtaaatttaacatgcaaacc |
| P_GAL1_-CYP-F | ctattacgccagctgaattgacatggcattaccaccatatacatatcca |
| P_GAL1_-CYP-R | caaacccaaaacaacagtccacattatagttttttctccttgacgttaaagtat |
| pESC-G418-F | caattcagctggcgtaatagcgaag |
| pESC-G418-R | atccgctctaaccgaaaaggaagg |
| AtCPR1- pESC-F | agcgtgacataactaattacatgattaccaaacatctctcaaatatctaccttcagt |
| AtCPR1- pESC-R | aaaagtaagaatttttgaaaattcaatataaatgacttctgctttgtatgcttctga |
| AtCPR2- pESC-F | aagcgtgacataactaattacatgattaccatacatctctaagatatcttccact |
| AtCPR2- pESC-R | aagaatttttgaaaattcaatataaatgtcctcttcttcttcttcgtca |
| CsCPR- pESC-F | gtaagcgtgacataactaattacatgatcaccacacatcacgcagatac |
| CsCPR- pESC-R | aaaaagtaagaatttttgaaaattcaatataaatgcaatcggaatccagttctatgaag |
| SgCPR2-pESC-F | gtgacataactaattacatgactaccaaacatctctcaaatatctaccagacat |
| SgCPR2-pESC-R | taagaatttttgaaaattcaatataaatgcaatctgaatctagatctatgaaagtttct |
| SrCPR1-pESC-F | aagcgtgacataactaattacatgattaattaagagcttaccaaacatctctcaag |
| SrCPR1-pESC-R | aagaatttttgaaaattcaatataaatgcaatctgattcagttaaagtttctcca |
| P_GAL1,10_- pESC -F | ttatattgaattttcaaaaattcttactttttttttgg |
| P_GAL1,10_- pESC -R | caaacccaaaacaacagtccacattatagttttttctccttgacgttaaagtat |
| T_CYC1_- pESC-F | ttcgctattacgccagctgaattgggccgcaaattaaagccttcg |
| T_CYC1_- pESC-R | tcatgtaattagttatgtcacgcttacattc |
| ERG1-F | atagcaatctaatctaagtttatgtctgctgttaacgttgcac |
| ERG1-R | gtgacataactaattacatgattaaccaatcaactcaccaaacaaaaatg |
| pY13- ERG1-F | aaacttagattagattgctatgctttct |
| pY13- ERG1-R | tcatgtaattagttatgtcacgcttacattc |
| AtCPR1-tRNA-CYP-F | tagccatcaaagattttggatgcgcaagcccggaatcgaa |
| AtCPR1-tRNA-CYP-R | aaggtagagctgttgaaatggttttagagctagaaatagcaagttaaaataaggct |
| pML104-Cas9-AtCPR1-CYP-F | catttcaacagctctaccttgatcatttatctttcactgcggagaagt |
| pML104-Cas9-AtCPR1-CYP-R | tccaaaatctttgatggctagttttagagctagaaatagcaagttaaaataaggct |
| pY15-GFP-F | tcatgtaattagttatgtcacgcttacattc |
| pY15-GFP-R | aaacttagattagattgctatgctttct |
| GFP -F | tgacataactaattacatgattatttgtacagttcatccatgccatg |
| GFP-Linker-R | ggaggcggtgggtccatgggtaagggagaagaacttttcact |
| CDS-Linker-GFP-R | cccatggacccaccgcctccttcagtcaaaactctatgacaatattcac |
| CDS-GFP-R | aaagcatagcaatctaatctaagtttatgtggagattgaaagttggtgct |
| EPH3-Linker-GFP-F | cccatggacccaccgcctccgaacttgttgatgaaatcatggatgtg |
| EPH3-GFP-R | atagcaatctaatctaagtttatggaccaaattgaacacattacca |
| CYP-Linker-GFP-F | cccatggacccaccgcctccttcttttggagtaaatttaacatgcaaacc |
| CYP-GFP-R | tagcaatctaatctaagtttatgtggactgttgttttgggtttg |
| AtCPR1-Linker-GFP-F | cttacccatgctgccgctgccgctaccccaaacatctctcaaatatctaccttcagttt |
| AtCPR1-GFP-R | gcaatctaatctaagtttatgacttctgctttgtatgcttctgatttg |
| T_ADH1_-mCherry-F | taaaaaaggagtagaaacattttgaagctatgagcgacctcatgctatacctgagaaa |
| T_ADH1_-mCherry-R | gcgaatttcttatgatttatgatttttattattaaat |
| SEC12-F | ataaatcataagaaattcgctcaagcatcatctatttctcgaaaagttgcg |
| SEC12-R | ggaagcggcggtggcggcagcatgtttttcaccaacttcatccttattgtgc |
| mCherry-F | gctgccgccaccgccgcttcccttgtacagctcgtccatgcc |
| mCherry-R | taaacacacataaacaaacaaaatggccatcatcaaggagttcatgc |
| P_GAP_-mCherry-F | tttgtttgtttatgtgtgtttattcgaaactaagttct |
| P_GAP_-mCherry-R | agggaacaaaagctggagctctcattatcaatactgccatttcaaagaatacg |
| pY15-mCherry-R | attctttgaaatggcagtattgataatgagagctccagcttttgttcccttt |
| **For genome integration** |  |
| 1622bU-F | gagccgcatcaatgctatcga |
| 1622bU-R | cgaaggctttaatttgcggcccgaactttacgaatcttgacgactacatga |
| T_CYC1_-EPH3-F | atttcatcaacaagttcgagctctagtcatgtaattagttatgtcacgcttacattcacg |
| T_CYC1_-EPH3-R | tcatgtagtcgtcaagattcgtaaagttcgggccgcaaattaaagcctt |
| EPH3-F | gtaagcgtgacataactaattacatgactagagctcgaacttgttgatgaaatcatgg |
| EPH3-R | ggggtaattaatcagcgaagcgatatggaccaaattgaacacattacca |
| P_GAL1,10_-EPH3-CDS-F | tgatggtaatgtgttcaatttggtccatatcgcttcgctgattaattacccc |
| P_GAL1,10_-EPH3-CDS-R | caactttcaatctccacatttatattgaattttcaaaaattcttactttttttttggat |
| SgCDs-F | aaaagtaagaatttttgaaaattcaatataaatgtggagattgaaagttggtgct |
| SgCDs-R | aataaaaatcataaatcataagaaattcgcttattcagtcaaaactctatgacaatattcac |
| T_ADH1_-CDS-F | atagagttttgactgaataagcgaatttcttatgatttatgatttttattattaaataag |
| T_ADH1_-CDS-R | aagcgtaatgtcgggagtgtgagcgacctcatgctatacctga |
| 1622bD-F | ctcaggtatagcatgaggtcgctcacactcccgacattacgctt |
| 1622bD-R | ccagcacaataccatataccaacg |
| 106a-U-F | gatattcctaagcctccctcacca |
| 106a-U-R | gacgctcgaaggctttaatttgcggcccacaaccgacgatccggg |
| 106a-D-F | cgctgtatagctcatatctttcccttcgctacccgaaagtttttccg |
| 106a-D-R | ctggcgttgtcaatgacagactt |
| T_CYC1_-AtCPR1-F | cccggatcgtcggttgtgggccgcaaattaaagccttcg |
| T_CYC1_-AtCPR1-R | gtagatatttgagagatgtttggtaatcatgtaattagttatgtcacgcttacattc |
| AtCPR1-F | agcgtgacataactaattacatgattaccaaacatctctcaaatatctaccttcagt |
| AtCPR1-R | aaaagtaagaatttttgaaaattcaatataaatgacttctgctttgtatgcttctga |
| P_GAL1,10_- AtCPR1-CYP-F | catacaaagcagaagtcatttatattgaattttcaaaaattcttactttttttttgg |
| P_GAL1,10_- AtCPR1-CYP-R | caaacccaaaacaacagtccacattatagttttttctccttgacgttaaagtat |
| CYP87D18-F | atactttaacgtcaaggagaaaaaactataatgtggactgttgttttgggtttg |
| CYP87D18-R | aatgcaagatttaaagtaaattcactttattcttttggagtaaatttaacatgcaaacc |
| T_TDH3_-CYP-F | gttaaatttactccaaaagaataaagtgaatttactttaaatcttgcatttaaataaat |
| T_TDH3_-CYP-R | tgccggaaaaactttcgggtagcgaagggaaagatatgagctatacagc |
| ΔCIT2-U-F | gtgcccgtaggtagagtaagt |
| ΔCIT2-U-R | tctttagatatggtgttcgttcctcacccctctgcatatttttctttcct |
| ΔCIT2-D-F | ggaaagaaaaatatgcagaggggtgaggaacgaacaccatatctaaag |
| ΔCIT2-D-R | cactccgactaatgtccttctaat |
| ΔMLS1-U-F | gcggcagtgacattgtctaatg |
| ΔMLS1-U-R | atatgtgtacactggggcaagggagaggtcatcgatccttcgcatt |
| ΔMLS1-D-F | aatgcgaaggatcgatgacctctcccttgccccagtgtaca |
| ΔMLS1-D-R | gcagaagaagaagctgtctggc |
| 308a-U-F | attgtgtgacaagaaagaacgga |
| 308a-U-R | ggatatgtatatggtggtaatgccatgtcacagcctatatctgttggtttgt |
| 308a-D-F | gcataatcatacattatcttttcaaagaataaacatggcatggcgatcagc |
| 308a-D-R | ctcaccgcatgacaagtggatc |
| 308a-dcas9-sgRNA-F | acaaaccaacagatataggctgtgacatggcattaccaccatatacatatcc |
| 308a-dcas9-sgRNA-R | ctgatcgccatgccatgtttattctttgaaaagataatgtatgattatgctttcactca |
| ΔGAL80-U-F | tcgcccgaacgacctcaaa |
| ΔGAL80-U-R | aaggagtagaaacattttgaagctatattgtgtgaccaaatgttgtgg |
| ΔGAL80-D-F | aaggctttaatttgcggcctggtcattgacattcacggtac |
| ΔGAL80-D-R | cagccttgcgtttatttcttgg |
| ΔGAL80-P_TEF1_-ERG1-T_CYC1_-F | ccacaacatttggtcacacaatatagcttcaaaatgtttctactcctt |
| ΔGAL80-P_TEF1_-ERG1-T_CYC1_-R | tctcccttggtaccgtgaatgtcaatgaccaggccgcaaattaaagcctt |
| 1021b-U-F | ttggtaacagaagatggcagtatttcca |
| 1021b-U-R | aaaggagtagaaacattttgaagctatggagatgcgacgaattactggc |
| 1021b-D-F | aaggctttaatttgcggccggcattatgagttaagagataatacgcacg |
| 1021b-D-R | gagaaaggacttaatccgtacacaatga |
| 1021b -P_TEF1_-ERG1-T_CYC1_-F | gccagtaattcgtcgcatctccatagcttcaaaatgtttctactcctt |
| 1021b -P_TEF1_-ERG1-T_CYC1_-R | cgtgcgtattatctcttaactcataatgccggccgcaaattaaagcctt |
| 208a-U-F | gctaaacatgccgtctccga |
| 208a-U-R | ctcgaaggctttaatttgcggccgagcactttacacagtgcaggaac |
| 208a-D-F | aaggagtagaaacattttgaagctatgatcacgacggcaatgacaaaaac |
| 208a-D-R | gaggcctgcacagacacttg |
| 208a -P_TEF1_-ERG1-T_CYC1_-F | gttcctgcactgtgtaaagtgctcggccgcaaattaaagccttcga |
| 208a -P_TEF1_-ERG1-T_CYC1_-F | gtttttgtcattgccgtcgtgatcatagcttcaaaatgtttctactcctt |
| 106a-CPRs-U-F | gatattcctaagcctccctcacca |
| 106a-CPRs-U-R | ttgggacgctcgaaggctttaatttgcggccgcattgacacacatctcaagtcatctc |
| 106a-CPRs-D-F | atttctggggtaattaatcagcgaagcgatcgctacccgaaagtttttccg |
| 106a-CPRs-D-R | ctggcgttgtcaatgacagactt |
| 106a-P_GAL10_-CPRs-T_CYC1_-F | ttatttgagatgacttgagatgtgtgtcaatgcggccgcaaattaaagccttcg |
| 106a-P_GAL10_-CPRs-T_CYC1_-R | ccatttagcttgccggaaaaactttcgggtagcgatcgcttcgctgattaattacc |


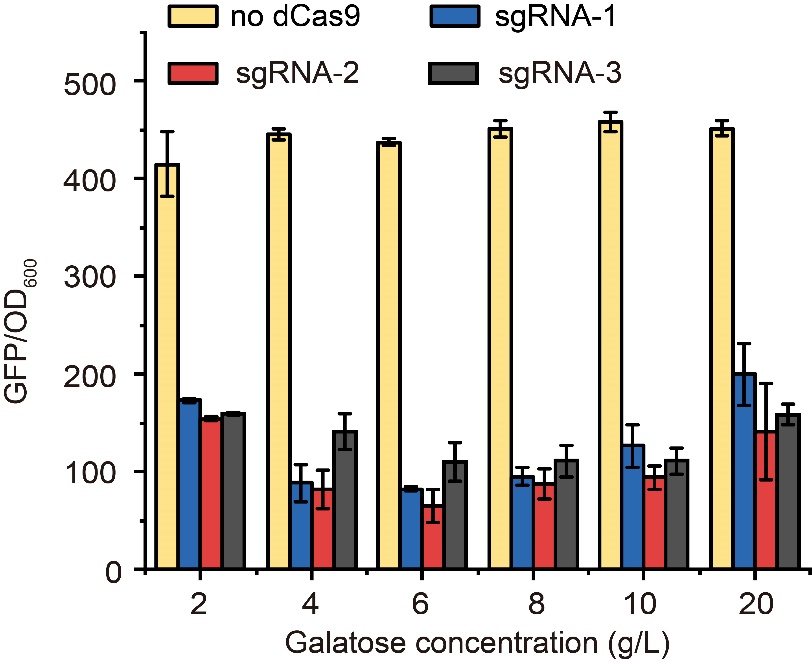


**Figure S1 The effect of sgRNA on GFP fluorescence without induction.** The dcas9 protein in the control group was not expressed in the absence of galactose. When adding inducer, the GFP/OD of control was significantly higher than those containing sgRNA. In addition, different concentrations of inducer have little effect on the value of GFP/OD in the control group. It suggested that the sgRNA did not affect the control level of CRISPRi.

***SgCDS* codon optimized sequence**

ATGTGGAGATTGAAAGTTGGTGCTGAATCTGTTGGTGAAAATGATGAAAAATGGTTGAAATCTATTTCTAATCATTTGGGTAGACAAGTTTGGGAATTTTGTCCTGATGCTGGTACTCAACAACAATTGTTGCAAGTTCACAAGGCTAGAAAAGCATTCCATGATGATAGATTTCACAGAAAACAATCCTCTGATTTGTTTATTACTATTCAATATGGTAAAGAAGTTGAAAATGGTGGTAAAACTGCTGGTGTTAAATTGAAAGAAGGTGAAGAAGTTAGAAAAGAAGCTGTTGAATCTTCTTTAGAAAGAGCTTTGTCTTTTTATTCTTCTATTCAAACTTCTGATGGTAATTGGGCTTCTGATTTGGGTGGTCCAATGTTTTTGTTGCCTGGTTTGGTTATTGCTTTGTATGTTACTGGGGTTTTGAACAGTGTGTTATCTAAACATCATAGACAAGAAATGTGTAGATACGTTTATAATCATCAAAATGAAGATGGGGGTTGGGGTTTGCATATTGAAGGTCCATCTACTATGTTTGGTTCTGCTTTGAATTATGTTGCTTTGAGATTGTTGGGTGAAGATGCTAATGCTGGTGCTATGCCAAAAGCTAGAGCTTGGATTTTGGATCATGGTGGTGCTACTGGTATTACTTCTTGGGGTAAATTGTGGTTGTCTGTTTTGGGTGTTTACGAATGGTCTGGTAATAATCCATTGCCACCTGAATTTTGGTTGTTTCCATATTTTTTGCCATTTCATCCTGGTAGAATGTGGTGTCATTGTAGAATGGTTTATTTGCCAATGTCTTATTTGTATGGTAAAAGATTTGTTGGTCCAATTACTCCAATTGTTTTGTCATTGAGGAAAGAACTATACGCTGTTCCGTATCATGAAATTGATTGGAATAAATCTAGAAATACTTGTGCTAAAGAAGATTTGTATTATCCACATCCAAAAATGCAAGATATTTTGTGGGGTTCTTTGCATCATGTTTATGAACCATTGTTTACTAGATGGCCTGCTAAAAGATTGAGAGAAAAAGCTTTGCAGACTGCTATGCAGCATATTCACTATGAAGACGAAAATACTAGATATATCTGCTTGGGTCCTGTTAATAAAGTTTTGAATTTGTTGTGTTGTTGGGTTGAAGATCCATATTCTGATGCTTTTAAATTGCATTTGCAAAGAGTTCATGATTACCTCTGGGTCGCTGAGGATGGTATGAAAATGCAAGGTTATAATGGTTCTCAATTGTGGGATACTGCTTTTTCTATTCAAGCTATTGTTTCTACTAAATTGGTTGATAATTATGGTCCAACTTTGAGAAAAGCACACGATTTTGTTAAATCTTCTCAAATTCAACAAGATTGTCCTGGTGATCCAAATGTTTGGTATAGACATATTCATAAAGGTGCTTGGCCATTTTCTACTAGAGATCATGGTTGGTTGATTTCTGATTGTACTGCTGAAGGTTTGAAAGCTGCTTTGATGTTGTCTAAATTGCCATCTGAAACTGTTGGTGAATCTTTGGAAAGAAATAGATTGTGTGATGCTGTTAATGTTTTGTTGTCTTTGCAAAATGATAATGGTGGTTTTGCTTCTTATGAATTGACTAGATCTTATCCATGGTTGGAATTGATTAATCCTGCTGAAACTTTTGGTGATATTGTTATTGATTATCCATATGTTGAATGTACTTCTGCTACTATGGAAGCTTTGACTTTGTTTAAAAAATTGCATCCTGGTCATAGAACTAAAGAAATTGATACTGCTATTGTTAGAGCTGCTAATTTTTTGGAAAATATGCAAAGAACTGATGGTTCTTGGTATGGTTGTTGGGGTGTTTGTTTTACTTATGCCGGTTGGTTTGGTATTAAAGGTTTGGTTGCTGCTGGTAGAACTTATAATAATTGTTTGGCTATTAGAAAAGCTTGTGATTTTTTGTTGTCTAAAGAATTGCCTGGTGGAGGTTGGGGTGAGTCTTATTTATCTTGTCAGAATAAAGTTTATACTAATTTGGAAGGTAATAGACCACATTTGGTTAATACTGCTTGGGTTTTGATGGCTTTGATTGAAGCTGGTCAAGCTGAAAGAGATCCAACTCCATTGCATAGAGCTGCTAGATTGTTGATTAATTCTCAATTGGAGAACGGTGATTTTCCTCAACAAGAAATAATGGGGGTATTTAATAAAAATTGTATGATTACCTATGCTGCTTATAGAAATATTTTTCCAATTTGGGCTTTGGGTGAATATTGTCATAGAGTTTTGACTGAATAA

***SgEPH3* codon optimized sequence**

ATGGACCAAATTGAACACATTACCATCAACACCAACGGTATCAAGATGCACATCGCCTCCGTTGGTACCGGTCCAGTCGTTTTGTTATTGCATGGCTTCCCAGAATTGTGGTACTCTTGGAGACACCAATTATTGTACTTGTCCTCTGTCGGTTACAGAGCCATTGCTCCAGATCTAAGAGGTTACGGTGACACCGATTCTCCAGCCTCCCCAACTTCTTACACCGCCTTACACATCGTCGGTGATTTGGTCGGTGCTTTGGACGAATTGGGTATCGAAAAGGTTTTCTTGGTTGGTCACGACTGGGGTGCTATCATTGCTTGGTACTTCTGTTTGTTTCGTCCAGACAGAATCAAGGCTTTGGTCAACTTGTCTGTTCAATTCATACCGAGAAACCCTGCTATTCCATTTATCGAAGGTTTCAGAACTGCTTTCGGTGATGACTTCTACATGTGTCGTTTCCAAGTCCCAGGTGAAGCTGAAGAAGACTTTGCTTCCATTGACACTGCCCAATTATTCAAGACTTCTTTGTGTAACAGATCATCTGCTCCACCATGCCTCCCAAAGGAAATTGGTTTCAGAGCTATTCCTCCACCAGAAAACCTACCATCCTGGTTGACTGAAGAAGATATCAATTATTACGCAGCTAAATTTAAGCAAACTGGTTTCACCGGTGCTTTGAACTACTATAGAGCTTTCGATTTGACCTGGGAATTAACAGCTCCATGGACTGGTGCTCAAATCCAAGTTCCAGTTAAATTCATTGTCGGTGACTCTGACTTGACTTACCATTTCCCAGGTGCTAAGGAATACATTCACAACGGTGGTTTCAAGAAGGATGTTCCATTGTTGGAAGAAGTTGTTGTCGTCAAGGACGCCTGTCACTTCATTAACCAAGAAAGACCACAAGAAATCAATGCTCACATCCATGATTTCATCAACAAGTTCGAGCTCTAG

***CYP87D18* codon optimized sequence**

ATGTGGACTGTTGTTTTGGGTTTGGCTACTTTGTTCGTTGCTTATTACATTCATTGGATCAATAAGTGGAGAGATTCTAAATTTAATGGTGTTTTGCCACCTGGTACTATGGGTTTGCCATTGATTGGTGAAACTATTCAATTGTCTAGACCATCTGATTCATTGGATGTTCATCCTTTTATTCAAAAAAAAGTTGAAAGATATGGTCCAATTTTTAAAACTTGTTTGGCTGGTAGACCTGTTGTCGTTTCTGCTGATGCTGAATTTAATAATTATATTATGTTGCAAGAAGGTAGAGCTGTTGAAATGTGGTATTTGGATACTTTGTCTAAATTTTTTGGTTTGGATACTGAATGGTTGAAGGCTTTGGGTTTGATTCATAAATATATTAGATCTATTACTTTGAATCATTTTGGTGCTGAAGCTTTGAGAGAAAGATTTTTGCCATTTATTGAAGCTTCTTCTATGGAAGCTTTGCATTCTTGGTCTACTCAACCATCTGTTGAAGTCAAAAACGCATCTGCTTTGATGGTTTTTAGAACTTCTGTCAATAAGATGTTCGGTGAAGATGCTAAAAAATTGTCTGGTAATATTCCTGGTAAATTTACTAAATTGTTGGGTGGTTTTTTGTCTTTGCCATTGAACTTTCCTGGTACTACTTATCATAAATGCTTGAAAGACATGAAAGAAATTCAAAAAAAATTAAGAGAAGTTGTTGATGATAGATTGGCTAATGTTGGTCCTGATGTTGAAGATTTTTTGGGTCAAGCATTTAAAGATAAGGAGTCTGAGAAATTTATCTCTGAAGAATTTATCATTCAGTTATTGTTTTCTATTTCTTTCGCTTCTTTCGAATCTATCTCAACTACTTTGACTTTGATTTTGAAATTGTTGGATGAACATCCTGAAGTTGTTAAAGAATTGGAAGTTGAACATGAAGCTATTAGAAAAGCTAGAGCTGATCCTGATGGTCCAATCACTTGGGAAGAATATAAATCTATGACTTTTACTTTGCAAGTTATTAATGAAACTTTGAGATTGGGTTCTGTTACTCCTGCTTTGTTGAGAAAAACTGTTAAAGATTTGCAAGTTAAAGGTAAAATTATTCCTGAAGGTTGGACTATTATGTTGGTTACTGCTTCTAGACATAGAGATCCAAAAGTTTATAAAGATCCACATATTTTTAATCCATGGAGATGGAAAGATTTGGATTCTATTACTATTCAAAAAAATTTTATGCCATTTGGTGGAGGTTTGAGACATTGTGCTGGTGCTGAATACTCAAAGGTTTATTTGTGTACATTCTTGCATATTTTGTGTACTAAATATAGATGGACTAAATTGGGTGGAGGTACTATTGCTAGAGCTCATATTTTGTCTTTTGAAGATGGTTTGCATGTTAAATTTACTCCAAAAGAATAA

***AtCPR1* codon optimized sequence**

ATGACTTCTGCTTTGTATGCTTCTGATTTGTTTAAACAATTGAAATCTATTATGGGTACTGATTCTTTGTCTGATGATGTTGTCTTGGTTATTGCTACTACTTCTTTGGCTTTGGTTGCTGGTTTTGTTGTTTTGTTGTGGAAAAAAACTACTGCTGATAGATCTGGTGAATTGAAACCATTGATGATTCCAAAATCTTTGATGGCTAAGGATGAAGATGACGATTTGGATTTGGGTTCTGGTAAAACTAGAGTTTCTATTTTTTTTGGTACTCAAACTGGTACTGCTGAAGGTTTTGCTAAAGCTTTGTCTGAAGAAATTAAAGCTAGATATGAAAAAGCAGCTGTTAAGGTTATTGATTTGGATGATTATGCTGCTGATGATGACCAATATGAGGAAAAATTGAAAAAAGAAACTTTGGCTTTTTTTTGTGTTGCTACTTATGGTGATGGTGAACCAACTGATAATGCTGCAAGGTTTTACAAATGGTTCACTGAAGAAAATGAAAGAGATATTAAATTGCAACAATTGGCTTACGGTGTTTTTGCTTTGGGAAACAGACAGTATGAACACTTTAATAAAATTGGTATTGTTTTGGATGAAGAATTGTGTAAAAAAGGTGCTAAAAGATTGATTGAAGTTGGTTTGGGTGATGACGATCAATCTATTGAAGATGATTTTAATGCTTGGAAAGAATCTTTGTGGTCTGAATTGGATAAATTGTTGAAGGACGAAGATGATAAATCTGTTGCTACTCCATATACTGCTGTTATTCCTGAATATAGAGTTGTTACTCATGATCCAAGATTTACTACTCAAAAATCTATGGAGTCAAATGTTGCTAATGGTAATACTACTATTGATATTCATCATCCATGTAGAGTTGATGTTGCTGTTCAAAAAGAATTGCATACTCATGAATCTGATAGGTCTTGTATTCATTTGGAATTCGATATTTCTAGAACTGGTATTACTTATGAAACTGGTGATCATGTTGGTGTTTATGCTGAAAATCATGTTGAAATTGTTGAAGAAGCTGGTAAATTGTTGGGTCATTCTTTGGATTTGGTTTTTTCTATTCATGCTGATAAAGAAGATGGTTCTCCATTGGAATCTGCTGTTCCACCTCCATTTCCTGGTCCATGTACTTTGGGTACTGGTTTGGCTAGATATGCTGATTTGTTGAATCCACCAAGAAAATCTGCTTTAGTCGCTTTGGCTGCTTATGCTACTGAACCATCTGAAGCTGAGAAGTTGAAACACTTGACTTCTCCTGATGGTAAAGATGAATATTCTCAATGGATTGTTGCTTCTCAAAGATCTTTGTTGGAAGTTATGGCTGCTTTTCCATCTGCTAAACCACCATTGGGTGTTTTTTTTGCTGCTATTGCTCCAAGATTGCAACCAAGATATTATTCTATTTCTTCATCTCCAAGATTGGCTCCATCTAGAGTTCATGTTACTTCAGCTTTGGTTTATGGTCCAACTCCAACTGGTAGAATTCATAAAGGTGTTTGTTCTACTTGGATGAAAAATGCTGTTCCTGCTGAAAAATCTCATGAATGTTCTGGTGCTCCAATTTTTATTAGAGCTTCTAATTTTAAATTGCCATCTAATCCATCTACTCCAATTGTTATGGTTGGTCCTGGTACTGGTTTAGCTCCATTTAGAGGTTTTTTGCAAGAAAGAATGGCTTTGAAAGAGGATGGTGAAGAATTGGGTTCATCTTTGTTATTCTTTGGTTGCAGAAATAGACAAATGGACTTTATCTATGAAGATGAATTAAACAATTTCGTTGATCAAGGTGTTATTTCTGAATTGATTATGGCTTTTTCTAGAGAAGGTGCTCAAAAAGAATATGTTCAACATAAAATGATGGAAAAAGCTGCTCAAGTTTGGGATTTGATTAAAGAAGAAGGTTATTTGTATGTTTGTGGTGATGCTAAAGGTATGGCTAGAGATGTTCATAGAACTTTGCATACTATTGTTCAAGAACAAGAAGGTGTTTCTTCATCTGAAGCAGAAGCTATTGTTAAAAAATTGCAAACTGAAGGTAGATATTTGAGAGATGTTTGGTAA

***AtCPR2* codon optimized sequence**

ATGTCATCTTCATCTTCATCTTCTACATCAATGATTGATTTGATGGCTGCTATTATTAAAGGTGAACCTGTTATTGTTTCTGATCCTGCTAATGCTTCTGCTTATGAATCTGTTGCTGCTGAATTGTCTTCTATGTTGATTGAAAATAGACAATTTGCTATGATTGTTACTACTTCTATTGCTGTTTTGATTGGTTGTATTGTTATGTTGGTTTGGAGAAGATCTGGTTCTGGTAATTCTAAAAGAGTTGAACCATTGAAACCATTGGTTATTAAACCAAGAGAAGAGGAAATTGATGATGGAAGGAAGAAAGTTACTATTTTTTTTGGTACTCAAACTGGTACTGCTGAAGGTTTTGCTAAAGCTTTGGGTGAAGAAGCTAAAGCTAGATATGAAAAAACTAGATTTAAAATTGTTGATTTGGATGATTATGCAGCAGACGATGATGAATATGAAGAAAAATTGAAAAAGGAAGATGTCGCTTTTTTCTTTTTGGCTACTTATGGTGATGGTGAACCAACTGATAATGCTGCTAGATTTTATAAATGGTTTACTGAAGGTAATGATAGAGGTGAATGGTTGAAAAACTTGAAATATGGTGTTTTCGGTTTGGGAAATAGACAATATGAACATTTTAACAAAGTTGCTAAAGTTGTTGATGATATTTTGGTTGAACAAGGTGCTCAAAGATTGGTTCAAGTTGGTTTGGGTGATGACGATCAATGTATTGAAGATGATTTTACTGCTTGGAGAGAAGCTTTGTGGCCTGAATTGGATACTATTTTGAGAGAAGAAGGTGATACTGCTGTTGCTACTCCATATACTGCTGCTGTTTTGGAGTATAGAGTTTCAATCCATGACTCTGAAGATGCTAAATTCAATGACATTAATATGGCTAATGGTAATGGTTATACTGTTTTTGATGCTCAACATCCATATAAAGCTAATGTTGCTGTTAAAAGAGAATTGCATACTCCTGAATCTGATAGATCTTGTATTCATTTGGAATTTGATATTGCTGGTTCTGGTTTGACTTATGAAACTGGTGATCATGTTGGTGTTTTGTGTGATAATTTGTCTGAAACTGTTGATGAAGCTTTGAGATTGTTGGATATGTCTCCTGATACTTATTTTTCTTTGCATGCTGAAAAAGAGGACGGAACTCCAATTTCTTCATCTTTGCCACCTCCATTTCCACCATGTAATTTGAGAACTGCTTTGACTAGATATGCTTGTTTGTTGTCTTCTCCAAAAAAATCTGCTTTGGTTGCTTTGGCTGCTCATGCTTCTGATCCAACTGAAGCTGAAAGATTGAAACATTTGGCTTCTCCTGCTGGTAAAGTTGATGAATATTCTAAATGGGTTGTTGAATCTCAAAGATCTTTGTTGGAAGTTATGGCTGAATTTCCATCTGCTAAACCACCATTGGGTGTTTTTTTTGCTGGTGTTGCTCCAAGATTGCAACCAAGATTTTATTCTATTTCTTCTTCACCTAAAATCGCTGAAACTAGAATTCATGTTACTTGTGCTTTGGTTTATGAAAAAATGCCAACTGGTAGAATTCATAAAGGTGTTTGTTCTACTTGGATGAAAAATGCTGTTCCATATGAAAAATCTGAAAATTGTTCTTCAGCACCAATCTTTGTTAGACAATCAAATTTTAAATTGCCATCTGATTCTAAAGTTCCAATTATTATGATTGGTCCTGGTACTGGTTTGGCTCCATTTAGAGGTTTTTTGCAAGAAAGATTGGCTTTGGTTGAGTCTGGTGTCGAATTGGGTCCATCTGTTTTGTTCTTTGGTTGTAGGAATAGAAGAATGGACTTTATTTATGAAGAGGAATTGCAAAGATTTGTTGAATCTGGTGCTTTGGCTGAATTATCTGTTGCTTTTTCTAGAGAAGGTCCAACTAAAGAATATGTTCAACATAAAATGATGGATAAAGCTTCTGATATTTGGAATATGATTTCTCAAGGTGCTTATTTGTATGTTTGTGGTGATGCTAAAGGTATGGCTAGAGATGTTCATAGATCTTTGCATACTATTGCTCAAGAACAAGGTTCTATGGATTCTACTAAAGCTGAGGGTTTTGTTAAAAATTTGCAAACTTCTGGTAGATATTTGAGAGATGTTTGGTAA

***CsCPR* codon optimized sequence**

ATGCAATCTGAATCTAGATCTATGAAAGTTTCTCCATTGGAATTGATGTCTGCTATTATTAGAAAAGCTATGGATCCATCTAGAGAATCTTCTGAATCTGTTAGAGAAGTCGCAACTTTGATTTTGGAAAATAGAGAATTTGTTATGATTTTGACTACTTTGTTGGCTGTTTTGATTGGTTGTGTTGTCGTTTTGGTTTGGAAAAGATCTTCTGGTCAAAAAGCTAAACCATTTGAACCACCAAAACAATTGATTGTTAAAGAACCTGAACCTGAAGTTGATGATGGTAAAAAGAAAGTTACTGTTTTTTTTGGTACTCAAACTGGTACTGCTGAAGGTTTTGCTAAAGCTTTGGCTGAAGAAGCTAAAGCTAGATATGAAAAAGCTACTTTTAGAGTTGTTGATTTGGACGACTACGCTGCAGATGACGATGAATATGAAGAAAAATTGAAAAAAGAAACTTTGGCTATTTTTTTTTTGGCTACTTATGGTGATGGTGAACCAACTGATAATGCTGCTAGATTTTATAAATGGTTTTCTGAAGGTAAAGAAAAAGGTGATTGGATTTCTAATTTGCAATATGCTGTTTTTGGTTTGGGTAATAGGCAATATGAACATTTTAACAAGATTGCTAAAGTTGTTGATGAACAATTGGCTGAACAAGGTGGTAAAAGATTGGTTCCTGTTGGTTTGGGTGATGACGATCAATGTATTGAAGATGATTTTTCTGCTTGGAGAGAAGCTTTGTGGCCTGAATTGGATAAATTGTTGAGAGATGACGATGATTCTACTACTGTTGCTACTCCATATACTGCTGCTGTTTTGGAATATAGAGTTGTTTTTTATGACGCTGCTGATGTTTCTGTTGAAGATAAAAGATGGGCTTTTGCTAATGGTCATGCTGTTTATGATGCTCAACATCCATGTAGAGCTAATGTTGCTATGAGAAAAGAATTGCATACTCCTGCTTCTGATAGATCTTGTATTCATTTGGAATTTGATATTTCTGGTACTGGTTTGACTTATGAAACTGGTGATCATGTTGGTGTTTTTTGTGAAAATTTGGATGAAACTGTTGAAGATGCTATTAGATTGATTGGTTTGTCTCCTGAAACTTATTTTTCTATTCATACTGATAAAGATGATGGTACTCCATTGGGTGGTTCTTCTTTGCCACCTCCATTTGCTCCATGTACTTTGAGAACTGCTTTGACTCAATATGCTGATTTGTTGTCTTCTCCAAAAAAATCTGCTTTGGTTGCTTTGGCTGCTCATGCTTCTGATCCTGCTGAAGCTGATAGATTGAGACATTTGTCATCTCCTGCTGGTAAAGATGAATATGCTCAATGGATTATTGCTTCTCAAAGATCTTTGTTGGAAGTTATGGCTGAATTTCCATCTGCTAAACCACCATTGGGTGTTTTTTTTGCTGCTGTTGCTCCAAGATTGCAACCAAGATATTATTCTATTTCTTCATCTCCAAGAATGGCTCCATCTAGAATTCATGTTACTTGTGCTTTGGTTTATGATAAAACTCCAACTGGTAGAATTCATAAAGGTGTTTGTTCTACTTGGATGAAAAATGCTGTTCCATTGGAAGAATCTCAAGCTTGTTCTTGGGCTCCAATTTATGTTAGACAATCTAATTTTAAATTGCCAACTGATTCTAAATTGCCAATTATTATGATTGGTCCTGGTACTGGTTTGGCTCCATTTAGAGGTTTTTTGCAAGAAAGATTGGCTTTGAAAGAAGCTGGTGTCGAATTGGGTCATTCTATTTTGTTTTTTGGTTGTAGAAATAGAAAGATGGACTACATTTATGAAGATGAATTGTCTAATTTTGCTGAAACTGGTGCTTTGTCTGAATTGATTGTTGCTTTTTCTAGAGAAGGTCCAACTAAAGAATATGTTCAACATAAAATGGTTGATAAAGCTTCTGATATTTGGAATATTTTGTCTCAAGGTGGTTATATTTATGTTTGTGGTGATGCTAAAGGTATGGCTAGAGATGTTCATAGAACTTTGCATAATATTGTTCAAGAACAAGGTTCTTTGGATTCTTCTAAAGCTGAATCTATGGTTAAAAATTTGCAAATGTCTGGTAGATATTTGAGAGATGTTTGGTAG

***SgCPR2* codon optimized sequence**

ATGCAATCTGAATCTAGATCTATGAAAGTTTCTCCATTGGAATTGATGTCTGCTATTATTAGAAAAGCTATGGATCCATCTAGAGAATCTTCTGAATCTGTTAGAGAAGTCGCAACTTTGATTTTGGAAAATAGAGAATTTGTTATGATTTTGACTACTTTGTTGGCTGTTTTGATTGGTTGTGTTGTCGTTTTGGTTTGGAAAAGATCTTCTGGTCAAAAAGCTAAACCATTTGAACCACCAAAACAATTGATTGTTAAAGAACCTGAACCTGAAGTTGATGATGGTAAAAAGAAAGTTACTGTTTTTTTTGGTACTCAAACTGGTACTGCTGAAGGTTTTGCTAAAGCTTTGGCTGAAGAAGCTAAAGCTAGATATGAAAAAGCTACTTTTAGAGTTGTTGATTTGGACGACTACGCTGCAGATGACGATGAATATGAAGAAAAATTGAAAAAAGAAACTTTGGCTATTTTTTTTTTGGCTACTTATGGTGATGGTGAACCAACTGATAATGCTGCTAGATTTTATAAATGGTTTTCTGAAGGTAAAGAAAAAGGTGATTGGATTTCTAATTTGCAATATGCTGTTTTTGGTTTGGGTAATAGGCAATATGAACATTTTAACAAGATTGCTAAAGTTGTTGATGAACAATTGGCTGAACAAGGTGGTAAAAGATTGGTTCCTGTTGGTTTGGGTGATGACGATCAATGTATTGAAGATGATTTTTCTGCTTGGAGAGAAGCTTTGTGGCCTGAATTGGATAAATTGTTGAGAGATGACGATGATTCTACTACTGTTGCTACTCCATATACTGCTGCTGTTTTGGAATATAGAGTTGTTTTTTATGACGCTGCTGATGTTTCTGTTGAAGATAAAAGATGGGCTTTTGCTAATGGTCATGCTGTTTATGATGCTCAACATCCATGTAGAGCTAATGTTGCTATGAGAAAAGAATTGCATACTCCTGCTTCTGATAGATCTTGTATTCATTTGGAATTTGATATTTCTGGTACTGGTTTGACTTATGAAACTGGTGATCATGTTGGTGTTTTTTGTGAAAATTTGGATGAAACTGTTGAAGATGCTATTAGATTGATTGGTTTGTCTCCTGAAACTTATTTTTCTATTCATACTGATAAAGATGATGGTACTCCATTGGGTGGTTCTTCTTTGCCACCTCCATTTGCTCCATGTACTTTGAGAACTGCTTTGACTCAATATGCTGATTTGTTGTCTTCTCCAAAAAAATCTGCTTTGGTTGCTTTGGCTGCTCATGCTTCTGATCCTGCTGAAGCTGATAGATTGAGACATTTGTCATCTCCTGCTGGTAAAGATGAATATGCTCAATGGATTATTGCTTCTCAAAGATCTTTGTTGGAAGTTATGGCTGAATTTCCATCTGCTAAACCACCATTGGGTGTTTTTTTTGCTGCTGTTGCTCCAAGATTGCAACCAAGATATTATTCTATTTCTTCATCTCCAAGAATGGCTCCATCTAGAATTCATGTTACTTGTGCTTTGGTTTATGATAAAACTCCAACTGGTAGAATTCATAAAGGTGTTTGTTCTACTTGGATGAAAAATGCTGTTCCATTGGAAGAATCTCAAGCTTGTTCTTGGGCTCCAATTTATGTTAGACAATCTAATTTTAAATTGCCAACTGATTCTAAATTGCCAATTATTATGATTGGTCCTGGTACTGGTTTGGCTCCATTTAGAGGTTTTTTGCAAGAAAGATTGGCTTTGAAAGAAGCTGGTGTCGAATTGGGTCATTCTATTTTGTTTTTTGGTTGTAGAAATAGAAAGATGGACTACATTTATGAAGATGAATTGTCTAATTTTGCTGAAACTGGTGCTTTGTCTGAATTGATTGTTGCTTTTTCTAGAGAAGGTCCAACTAAAGAATATGTTCAACATAAAATGGTTGATAAAGCTTCTGATATTTGGAATATTTTGTCTCAAGGTGGTTATATTTATGTTTGTGGTGATGCTAAAGGTATGGCTAGAGATGTTCATAGAACTTTGCATAATATTGTTCAAGAACAAGGTTCTTTGGATTCTTCTAAAGCTGAATCTATGGTTAAAAATTTGCAAATGTCTGGTAGATATTTGAGAGATGTTTGGTAG

***SrCPR1* codon optimized sequence**

ATGCAATCTGATTCAGTTAAAGTTTCTCCATTCGATTTGGTTTCAGCTGCAATGAACGGTAAAGCTATGGAAAAGTTGAACGCATCTGAATCAGAAGATCCAACTACATTGCCAGCTTTGAAGATGTTGGTTGAAAACAGAGAATTGTTAACTTTGTTTACTACATCTTTCGCTGTTTTGATCGGTTGTTTGGTTTTCTTGATGTGGAGAAGATCTTCATCTAAGAAATTGGTTCAAGATCCAGTTCCACAAGTTATTGTTGTTAAAAAGAAAGAAAAGGAATCTGAAGTTGATGATGGTAAAAAGAAAGTTTCTATTTTCTATGGTACTCAAACAGGTACTGCAGAAGGTTTTGCTAAAGCATTGGTTGAAGAAGCTAAAGTTAGATACGAAAAGACTTCTTTTAAAGTTATTGATTTGGATGATTATGCTGCAGATGATGATGAATACGAAGAAAAGTTGAAGAAAGAATCATTGGCATTTTTCTTTTTGGCAACATATGGTGACGGTGAACCAACTGATAACGCTGCAAACTTCTACAAGTGGTTCACAGAAGGTGACGATAAGGGTGAATGGTTGAAGAAATTGCAATATGGTGTTTTTGGTTTGGGTAACAGACAATACGAACATTTCAATAAGATTGCAATTGTTGTTGATGATAAGTTGACTGAAATGGGTGCTAAGAGATTGGTTCCAGTTGGTTTAGGTGACGATGATCAATGTATCGAAGATGATTTCACAGCTTGGAAAGAATTGGTTTGGCCAGAATTGGATCAATTGTTGAGAGATGAAGATGATACATCTGTTACTACACCATATACTGCTGCAGTTTTGGAATACAGAGTTGTTTACCATGATAAACCAGCTGATTCATACGCAGAAGATCAAACACATACTAATGGTCATGTTGTTCATGATGCACAACATCCATCTAGATCAAACGTTGCTTTTAAGAAAGAATTGCATACATCTCAATCAGATAGATCTTGTACTCATTTGGAATTCGATATCTCTCATACAGGTTTATCATATGAAACTGGTGACCATGTTGGTGTTTACTCTGAAAATTTGTCAGAAGTTGTTGATGAAGCTTTGAAGTTGTTGGGTTTATCTCCAGATACATACTTCTCAGTTCATGCAGATAAGGAAGATGGTACTCCAATTGGTGGTGCTTCTTTGCCACCACCATTTCCACCATGTACATTAAGAGATGCTTTGACTAGATACGCAGATGTTTTGTCATCTCCAAAGAAAGTTGCTTTGTTAGCATTAGCTGCACATGCTTCTGATCCATCAGAAGCAGATAGATTGAAGTTCTTGGCTTCTCCAGCTGGTAAAGATGAATATGCTCAATGGATCGTTGCAAACCAAAGATCATTGTTGGAAGTTATGCAATCTTTTCCATCAGCAAAACCACCATTGGGTGTTTTCTTTGCTGCAGTTGCTCCAAGATTGCAACCAAGATACTACTCTATCTCATCTTCACCAAAGATGTCACCAAACAGAATCCATGTTACTTGTGCATTGGTTTACGAAACTACACCAGCTGGTAGAATTCATAGAGGTTTGTGTTCTACATGGATGAAAAATGCAGTTCCATTAACTGAATCACCAGATTGTTCTCAAGCTTCAATCTTCGTTAGAACATCTAACTTCAGATTGCCAGTTGATCCAAAAGTTCCAGTTATTATGATTGGTCCAGGTACTGGTTTGGCACCTTTTAGAGGTTTCTTGCAAGAAAGATTGGCTTTAAAAGAATCTGGTACAGAATTGGGTTCTTCAATTTTCTTTTTCGGTTGTAGAAACAGAAAAGTTGATTTCATCTATGAAGATGAATTGAACAACTTTGTTGAAACAGGTGCTTTGTCTGAATTGATCGTTGCATTTTCAAGAGAAGGTACTGCTAAGGAATACGTTCAACATAAGATGTCTCAAAAGGCATCAGATATCTGGAAGTTGTTGTCTGAAGGTGCTTATTTGTACGTTTGTGGTGACGCTAAGGGTATGGCAAAGGATGTTCATAGAACATTGCATACTATTGTTCAAGAACAAGGTTCTTTAGATTCTTCAAAGGCTGAATTGTACGTTAAAAATTTGCAAATGTCAGGTAGATACTTGAGAGATGTTTGGTAAGCTCTTAATTAA
